# Optimized collusion prevention for online exams during social distancing

**DOI:** 10.1038/s41539-020-00083-3

**Published:** 2021-03-01

**Authors:** Mengzhou Li, Lei Luo, Sujoy Sikdar, Navid Ibtehaj Nizam, Shan Gao, Hongming Shan, Melanie Kruger, Uwe Kruger, Hisham Mohamed, Lirong Xia, Ge Wang

**Affiliations:** 1 Department of Biomedical Engineering, Rensselaer Polytechnic Institute, Troy, NY USA; 2 Department of Computer Science, Rensselaer Polytechnic Institute, Troy, NY USA; 3 Department of Computer Science and Engineering, Washington University in St. Louis, St. Louis, MO USA; 4 Department of Mechanical Aerospace and Nuclear Engineering, Rensselaer Polytechnic Institute, Troy, NY USA

**Keywords:** Education, Interdisciplinary studies, Institutions, Education

## Abstract

Online education is important in the COVID-19 pandemic, but online exam at individual homes invites students to cheat in various ways, especially collusion. While physical proctoring is impossible during social distancing, online proctoring is costly, compromises privacy, and can lead to prevailing collusion. Here we develop an optimization-based anti-collusion approach for distanced online testing (DOT) by minimizing the collusion gain, which can be coupled with other techniques for cheating prevention. With prior knowledge of student competences, our DOT technology optimizes sequences of questions and assigns them to students in synchronized time slots, reducing the collusion gain by 2–3 orders of magnitude relative to the conventional exam in which students receive their common questions simultaneously. Our DOT theory allows control of the collusion gain to a sufficiently low level. Our recent final exam in the DOT format has been successful, as evidenced by statistical tests and a post-exam survey.

## Introduction

Testing is essential for measuring and improving educational outcomes^1^, but a major concern is that many students tend to cheat^2,3^. As suggested in a study between 2002 and 2015 by Dr. McCabe and the International Center for Academic Integrity^4^, cheating among students was found astonishingly prevailing, e.g., 43%, 68%, and 95% of graduate students, undergraduate students, and high school students, respectively, admitted to cheating in assignments or exams.

Recently, the cheating problem has become much worse. In response to the COVID-19 pandemic, online learning has become necessary and exclusive in most educational systems^5,6^. The hard landing from the conventional education environment to the “emergency” online learning mode^7^ creates various challenges, such as limited access to resources^8^, lack of experience/skills^9,10^, concerns over the quality and efficacy of education^6,11^, as well as exacerbation of educational inequality^12^. As far as the assessment of learning outcomes is concerned, social distancing works directly against proctoring^13^ since online testing performed at individual homes simply creates more chances to cheat^14^ and increases temptation to do so^15–17^. Traditionally, physical invigilation is routinely used to suppress cheating. How to proctor online exams presents a new challenge during social distancing^6^, as conventional approaches do not take the pandemic into account^14^. Rigorous online proctoring methods with cameras and associated technologies have been designed and used to prevent cheating^18^ during the pandemic to effectively improve learning outcomes^19,20^. Professional services exist for online proctoring, such as TOP HAT^21^ (used by over 400 institutions), Examity^22^ and Proctortrack^TM^^23^ (proctored over two million exams). They monitor students through webcams and screen videos, enforce a full screen mode, and disable any content sharing. Some proctoring companies sign contracts with schools, while others charge students instead; as examples, ProctorU charges students $15 per test, while Proctorio charges a $100 lifetime fee. In addition to the costs associated with the use of third-party proctoring software, there are concerns over privacy^24–26^. What aggravates the problem of cheating is the “digital arms race”, i.e., “finding new ways of cheating requires new ways to prevent it”^27^.

Despite the benefit of rigorous proctoring, there is also a valid concern that using “such draconian measures” bluntly signals to our students the lack of our trust in their honesty^14^. Hence, in contrast to control the remote assessment environment, the OpenProctor system has been developed recently which extracts the writing style from learner-generated data and utilizes it as a behavioral biometrics to validate the authorship of students with machine learning^28^. This method demonstrated a mean accuracy of 93% significantly higher than the human performance baseline of 12%^29^. Unfortunately, the utility of this type of method is limited to text plagiarism and does not apply to multiple-choice and calculation questions, which are necessary and essential in majority science and engineering courses^15^. As mentioned in ref. ^30^, due to the highly objective nature of “math or fact-based” courses, it is more challenging and frequently questionable to maintain academic integrity without proctoring compared to the subjective “writing-based” courses. In addition, this writing-style recognition method mainly focuses on the post-exam stage, which may not be enough since it does not reduce the practicality of cheating and is not optimal as questioned by Fuller et al.^14^. (“Is Faculty’s role to merely catch and punish cheating students or is it to support students through their studies so that ultimately, they can be confident that by working hard they will be successful without having to resort to deception?”)

Besides such fancy techniques, traditional online learning experience also offers tips and recommendations without the use of cameras, which can be integrated to form a practical solution; e.g., sequencing questions randomly, presenting questions in limited time slots^31^, and drawing assessment questions from a large pool^26,32^.

However, the transition from an emergency ad hoc remote assessment^7^ to a valid conventional online assessment requires extensive efforts from educators; e.g., creating a large question pool. The pool size often needs to be huge to make the overlap of questions negligible between tests, e.g., a 300-question pool is needed for 30-question tests to control the average number of questions in common for two students below 3 (square of the number of questions in a test divided by the pool size)^15,26^. Such a pool is so large that is impractical to be updated frequently, which makes it vulnerable to cheating as evidenced by the rapid growth of exam questions being posted online during the pandemic.

Here we address the above limitations by providing an optimization-based cost-effective and privacy-conserving solution to help educators perform a valid remote assessment with minimal efforts. Specifically, the following three features of our approach are underlined: First, our method is optimization-based for simultaneously minimizing the productivity and practicality of cheating. The two models of the decision-making process behind cheating are well known: ref. ^33^ proposed a model involving the two competing processes of rational cost–benefit and effects on “self-concept", and ref. ^34^ developed a model based on the fraud triangle of incentive/pressure, rationalization, and opportunity. Our approach substantially increases the cost–benefit ratio and decreases the opportunity, and hence directly guides students to realize that it is more productive to finish the exam independently than to cheat. This curb on collusion is independent of proctoring, and respectful to privacy. Second, our method minimizes the question pool size; e.g., a pool of size 1.5 times the number of questions in a test is found to be sufficient to suppress collusion gain to an insignificant level with our method. The substantially smaller required pool size allows educators to devise their own questions relatively easily that require intellectual efforts than factual recalls that can be simply done via Google search^14^, and update the questions frequently^31^ rather than directly rely on published question banks without paraphrasing^32^ which has a high risk of inviting academic dishonest^35–37^. Clearly, our framework encourages better compliance with best practices because of smaller question banks. Third, our method mainly focuses on thwarting collusion, which is believed to be significantly more popular than other types of cheating behaviors in online exams as found in a survey study based on self-reports^17^ and validated later by direct measurements^38^, showing that about 80% cheating events belonged to collusion, 42% showed copying from Internet website, and 21% fell into both categories. Other types of misconduct, such as accessing unauthorized sources and contract cheating, may also exist which can be addressed by incorporating readily available techniques; e.g., design open books questions^39–41^, profile based authentication^42^, challenging questions^43,44^, and Web video conference proctoring.

**Fig. 1:**
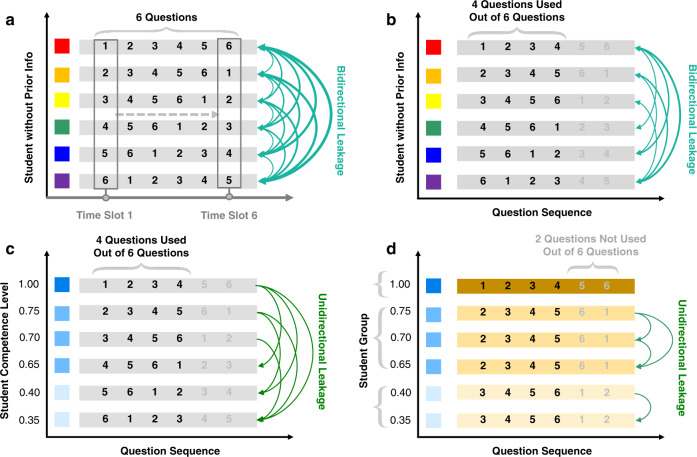
Anti-collusion mechanisms with and without prior knowledge of students’ competencies. Assume that collusion happens between two students and one can get answers from the other on questions that the other has already answered or is working on (see “Methods”). **a** The circulation-based scheme is illustrated with a simple example, in which six students take an exam consisting of six questions (*M*_1_ = *M*_2_ = 6) provided to each student one by one, and each question must be finished within the allocated time slot shown as the vertical box. If there is no information of students' competences, this scheme helps reduce potential bidirectional cheating among students to ~50% of question; **b** the collusion chance can be made even less if cheating students are fed with more new questions (*M*_1_ = 4, *M*_2_ = 6); **c** if prior information on students' competences is available, the naive assignment in **b** still yields significant collusion gains; but **d**, using our grouping-based anti-collusion scheme, the maximum and average collusion gains can be sharply reduced to ~10% and ~3%, respectively. The scheme first divides the competence range into *M*_2_ − *M*_1_ + 1 intervals, then groups the students into these intervals properly, finally assigns these groups of students with the corresponding number of consecutive cyclic sequences, respectively. The maximum collusion gain with this scheme is bounded by our Theorem 1.

In the following, we will focus on the key elements of our approach although the aforementioned complementary strategies are also important to complement our approach into an integrated practical solution to the anti-cheating problem. Our method is mainly designed for “math or fact-based” courses and compatible with most types of questions, and here is illustrated with a multiple-choice question (MCQ)-based model, since MCQs are popular, reliable, valid, and cost-effective^45,46^. Our main results are a theorem giving an upper bound of the collusion gain for our exam design, scheduling algorithms for anti-collusion in our distanced online testing (DOT) platform, and our DOT exam results. Using our DOT technology, the collusion gain can be practically and theoretically made insignificant, especially by incorporating prior knowledge of the students’ competences. The collusion gain refers to the percentage score increased by a student through collusion, and competence represents the student’s individual probability by which he/she can correctly answer questions in an exam. Our main idea is to optimally deliver questions to students as individual-specific sequences in a synchronized fashion so that even if students freely cheat among themselves they still cannot significantly improve their scores (Fig. 1).

## Results

### Theorem bounding the collusion gain

As a first order of approximation, our analysis is focused on an idealized DOT scenario, but our analysis can be extended to more general settings without theoretical or technical difficulties. In our initial DOT setting, *M*_1_ MCQs from a pool of *M*_2_ MCQs (for example, with equal difficulty and credits for convenience, which can be readily relaxed for a more accurate analysis) are provided to a class of *N* students, and there are *Q* choices per question with one being correct. All *N* students are presented with their own set of *M*_1_ questions displayed one by one in generally different *sequences*, and are asked to take the exam simultaneously. Each student must answer each question in a predetermined time slot, and cannot revisit previous questions. This mode of delivering questions is exemplified in Fig. 1a.

Under practical assumptions on students’ collusion behaviors (“Methods”), we propose a grouping-based anti-collusion scheme (GAS) to control the collusion gain below any desired level with prior knowledge on students’ competences. The competence of a student can be easily estimated based on his/her grade point average (GPA) (rough surrogates), from earlier quizzes (better indicators), and/or with a first portion of the exam (achievable via dynamic programming). Generally speaking, our grouping-based approach consists of the following three elements: (1) *Grouping*: Students with similar competences are grouped together to receive the same sequence of questions in an exam; (2) *Optimization*: The number of questions that can be copied between groups is aggressively reduced (even to zero coupled with the next element); (3) *Augmentation*: The pool of questions can be enlarged to have the number of questions greater than *M*_1_.

The anti-collusion exam design can efficiently reduce the collusion gain mainly due to following reasons (Fig. 1b–d): (1) The maximum question leakage from top to down of *C* consecutive cyclic sequences can be reduced to zero if *M*_2_ − *M*_1_ + 1 ≥ *C* (Supplementary Fig. 1); (2) by grouping, the equivalent number of students (the number of groups) can be significantly reduced to just use the *C* sequences; (3) students with similar competences have small probabilities to cheat within their group due to the fact that they can only obtain tiny collusion gains, although the intra-group collusion is facilitated because of the same sequence shared. With this procedure, by making *C* = *M*_2_ − *M*_1_ + 1 sufficiently large we can control the maximum individual collusion gain as well as the average collusion gain below any desired level.

Mathematically, we present the following theorem that shows the upper bound of the collusion gain associated with our GAS (Supplementary Note 1).

#### Theorem 1

*Given sequences of* *M*_1_ *questions from the bank of* *M*_2_ MCQs *with one and only one correct choice out of Q choices for each question, the maximum individual collusion gain can be controlled to be no larger than (1* *−* *1/Q)/(M*_*2*_ *−* *M*_*1*_ *+* *1) using the GAS*.

This theorem is practically powerful; e.g., according to this upper bound, the maximum individual collusion gain can be theoretically controlled below 3.6% for any large-size class with a reasonable test setting of *M*_2_ = 60, *M*_1_ = 40, *Q* = 4.

### Metrics characterizing the final exam design

Our aforementioned theorem provides an upper bound for collusion control, but it is usually not optimal since it does not fully take advantage of the knowledge of students’ competences. Based on the results of GAS, discrete optimization algorithms (“Methods”) can be used to further reduce the collusion gain for the best DOT anti-collusion performance. For this purpose, the objective function needs to be defined as follows.

Let us introduce the *competence profile* of students *Y* = {*y*_*i*_ ∈ [1/*Q*, 1]∣ *i* = 1, 2, …, *N*} in a non-increasing order, and a *colluding matrix* $P={({p}_{j,i})}_{i,j\in [N]}$, where *p*_*j*,*i*_ represents the probability of student *i* colluding from student *j* if *i* ≠ *j*, and *p*_*i*_,_*i*_ the probability that student *i* does not cheat in the exam. *P* is upper triangular. Given an *assignment* *A* = (*a*_*i*_, …, *a*_*N*_) which is a vector whose elements are sequences of questions (SQs), where *a*_*i*_ is the SQ assigned to student *i*, the average collusion gain *g* is the total collusion gain normalized with respect to the class size and the number of questions in an exam, and defined as

1 $$g(A)=\frac{\,{\text{sum}}\,\{Z(A)\circ P\circ D\}}{N{M}_{1}}=\mathop{\sum }\limits_{i = 1}^{N}\mathop{\sum }\limits_{j = 1}^{i-1}\frac{{z}_{j,i}(A)}{N{M}_{1}}{p}_{j,i}({y}_{j}-{y}_{i})$$

where sum{⋅} stands for the operation of summing up all elements, ∘ denotes the Hadamard (element-wise) multiplication, the *competence difference matrix* *D* is defined as ${({d}_{j,i})}_{i,j\in [N]}$ where ${d}_{j,i}=\max ({y}_{j}-{y}_{i},0)$, and the *positional matrix*$Z={({z}_{j,i})}_{i,j\in [N]}$ is determined by *A* where *z*_*j*,*i*_ represents the number of questions that student *i* can cheat from student *j* if *j* ≠ *i*, and the special case *z*_*i*,*i*_ is defined as *M*_1_. If all students use the same SQ as in the conventional exam scenario without collusion prevention, the average collusion gain becomes

2 $${g}_{0}=\frac{\,{\text{sum}}\,\{P\circ D\}}{N}=\frac{1}{N}\mathop{\sum }\limits_{i = 1}^{N}\mathop{\sum }\limits_{j = 1}^{i-1}{p}_{j,i}({y}_{j}-{y}_{i}).$$

We developed our DOT platform (Supplementary Note 6) incorporating the anti-collusion techniques as well as other complementary techniques for online exams, and applied this platform for the final exam of an undergraduate imaging course on 28 April 2020. Totally, 78 out of 85 undergraduate students took the exam from two separately taught classes. The exam consisted of *M*_1_ = 40 questions that were assigned to each student and scheduled from a pool of *M*_2_ = 60 questions by applying our greedy algorithms with a heuristically constructed colluding matrix *P*, detailed in the “Methods” section and Supplementary Note 4. During the exam, the students were asked to join a WebEx session for the instructors to address any questions or technical difficulties (in principle, our DOT technology can be combined with sounds online proctoring for an enhanced performance at an additional cost). The competency information of the students was estimated based on their performance in the midterm exam conducted before the class was taught online.

The optimized assignments led to orders of magnitude reduction in the collusion gain. Quantitatively, the average collusion gain was reduced to 0.0073% from 19.23% (a reduction by three orders of magnitude from the conventional scenario), with the worst-case collusion gain (*g*_W_, the average collusion gain when every student manages to achieve his/her maximum possible collusion gain; see “Methods”) and the maximum individual collusion gain (*g*_MI_, the maximum of the maximum possible collusion gains over all students; see “Methods”) being 0.91% and 6.88%, respectively. Specifically, we performed numerical simulation to estimate the average collusion gain with optimized assignments under the following conditions: (1) accurate *Y* and random *P*, the estimated *Y* was assumed to be faithful and the colluding probabilities *p*_*k,i*_ (*k* < *i*, *i* − 1 in total) assumed to follow the (*i* − 1)-variate *Dirichlet* distribution with a concentration parameter of *α* = 10; (2) noisy *Y* and random *P*, the estimated *Y* was assumed to contain a Gaussian noise (*μ* = 0, *σ* = 0.05). We calculated the average collusion gains *g* as well as the worst-case metrics *g*_W_ and *g*_MI_ with the same assignments (the conventional scenario) and with our optimized assignments over 500 instances for each condition. The resultant means and standard deviations demonstrate the accuracy and robustness of our DOT technology (Table 1a).

**Table 1:** Collusion gain estimation and optimization in the case of *N* = 85, *M*_2_ = 60, *M*_1_ = 40, and *Q* = 4.

Condition	Anti-collusion	Mean (standard seviation)		
		*g*	*g*_W_	*g*_MI_
(a) Collusion gain estimation of the optimized assignment (500 instances)^a^				
Accurate *Y*, random *P*	None	0.14497 (0.00139)	0.30901 (–)	0.75000 (–)
	Optimized	**0.00044** (0.00005)	**0.00908** (–)	**0.06878** (–)
Noisy *Y*, random *P*	None	0.14884 (0.00450)	0.30837 (0.00708)	0.73524 (0.02107)
	Optimized	**0.00190** (0.00052)	**0.06275** (0.00775)	**0.20404** (0.02941)
(b) Optimized performance over 500 random *Y* profiles^b^				
Random *Y*, heuristic *P*	None	0.16278 (0.01499)	0.30384 (0.04153)	0.60838 (0.06344)
	Optimized	**0.00007** (0.00002)	**0.00903** (0.00158)	**0.04970** (0.01650)
(c) Optimizations of different class sizes over 500 random *Y* profiles with heuristic *P*^c^				
*N* = 20, *M*_2_ = 30, *M*_1_ = 20	Optimized	0.00013 (0.00007)	0.00657 (0.00223)	0.03704 (0.01812)
*N* = 40, *M*_2_ = 60, *M*_1_ = 40	Optimized	0.00003 (0.00002)	0.00433 (0.00130)	0.02686 (0.01356)
*N* = 100, *M*_2_ = 60, *M*_1_ = 40	Optimized	0.00008 (0.00002)	0.00936 (0.00139)	0.04847 (0.01777)
*N* = 500, *M*_2_ = 60, *M*_1_ = 40	Optimized	0.00011 (0.00001)	0.01400 (0.00086)	0.07886 (0.01629)

^a^Robustness of the optimized assignments: The collusion gain of assignments optimized with the heuristic *P* in which the colluding probability is proportional to the competence difference between two students (see “Methods”) is reproduced in two kinds of perturbations: noisy *Y* (Gaussian noise (*μ* = 0, *σ* = 0.05) on accurate *Y*) and *P* variations (random colluding probabilities following the Dirichlet distribution). ^b^Stability of the optimized performance: The optimization results over 500 random *Y* profiles, each of which was randomly generated according to a Gaussian distribution (*μ*_0_ = (1 + 1/*Q*)/2 and *σ*_0_ = (1 − 1/*Q*)/6 on the support [1/*Q*, 1]). ^c^Optimization performances on small-size classes (*N* = 20, *M*_2_ = 30, *M*_1_ = 20, and *Q* = 4), middle-small-size classes (*N* = 40, *M*_2_ = 60, *M*_1_ = 40, and *Q* = 4), middle-size classes (*N* = 100, *M*_2_ = 60, *M*_1_ = 40, and *Q* = 4), and large-size classes (*N* = 500, *M*_2_ = 60, *M*_1_ = 40, and *Q* = 4). Bold indicates the better result.

To further illustrate the performance of our DOT technology, in the setting of the above practical case we performed numerical simulations assuming random Gaussian-distributed competence profiles with *μ*_0_ = (1 + 1/*Q*)/2 and *σ*_0_ = (1 − 1/*Q*)/6, truncated to be meaningful [1/*Q*, 1] and heuristically constructed *P* from *Y*. We calculated the collusion gains without collusion prevention *g*_0_ and with optimized prevention *g* over 500 instances for each configuration. Our results are summarized in Table 1b. It can be observed in this case that the mean of the average collusion gain can be reduced by three orders of magnitude with tiny standard deviations, suggesting that our DOT designs are not only effective but also stable in controlling the collusion gain. We further changed the number of students to those of four typical class sizes from *N* = 20 to *N* = 500 as shown in Table 1c, and the mean of the average collusion gain remains at a very small level which implies the practical applicability of our method in dealing with a wide range of class sizes.

### Analyses on the final exam

**Fig. 2:**
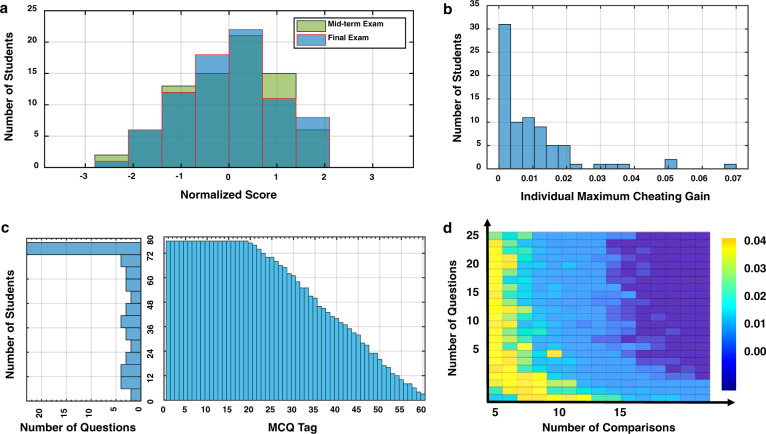
Results of the final exam. **a** The distribution of the final exam scores (78 students, conducted on the DOT platform) in a normal ‘bell' shape with insignificant differences from the distribution of the midterm exam scores (before social distancing), which indicates the effectiveness of our DOT technology in collusion prevention. **b** With our optimized question assignment, the distribution of calculated maximum possible collusion gains in terms of percentage score showing the maximum collusion gains strictly below 7%. **c** The histogram of the size of a particular set of MCQs versus the number of students who received this set of questions on the left, and the distribution of this number versus the MCQ tag on the right, showing that not all student received the same questions. **d** Plotting *q* values against the number of comparisons and the number of questions to test the hypothesis that collusion resulted in students giving a significantly higher number of the same incorrect answers. As all *q* values are below the significance level of 0.05, we conclude that no significant collusion occurred.

We first look at the final exam results, which are summarized in several histograms. The normalized distribution (zero mean, unit std.) of the 78 students’ scores out of 40 questions is, as expected, an approximate “bell-shaped curve” of a normal distribution (Fig. 2a). As a first comparison by eye, we contrast the distribution of the final exam results with that of the midterm exam, which serves as a control group here. For a more quantitative analysis, we applied standard tests to the results of the midterm and final exams to ascertain whether there are any anomalies embedded within the two sets. First, we found that both sample sets were drawn from normal distributions by applying the Anderson–Darling test^47^ (*p* = 0.1570 and *p* = 0.3004 for the midterm and final samples, respectively). Next, we confirmed that both sample sets were drawn from the same normal distribution using the two-sample Kolmogorov–Smirnov test^48^ (*p* = 0.1574). As an additional test, we applied the two-sample *t*-test for equal variance^49^ and confirmed that the two distributions have the same mean (*p* = 0.7997). In summary, the evidence does not support the claim that there are differences in the distributions of the midterm and the final exam, demonstrating consistent evaluative results of the same population between the conventional physical proctoring method (the midterm) and our DOT format (the final).

Quantitatively, the maximum gain of the students through collusion is theoretically controlled by design to be below 7% (Fig. 2b). This compares favorably to a maximum gain of 75% without the use of our optimized anti-collusion technique. It is important to note that over 90% of students may have a maximum collusion gain of below 2%, which underpins the effectiveness of our technique. One feature of this technique is that not all student shared the same question sets, which helped to reduce the colluding chances between students. In terms of the number of recipients of each MCQ, only 19 questions were assigned to all students, and 20 questions were assigned to fewer than 40 students each (Fig. 2c).

Following from the preceding discussion, utilizing our anti-collusion exam design the controlled collusion gain was made very small but is still not zero. It is therefore imperative to test whether significant collusion did occur. To do so, we examined the following two aspects: (i) what is the frequency with which pairs of students gave the same incorrect answer and (ii) is the average number of correct answers to the first 20 questions comparable to that of the last 20 questions. The rationale for aspect (i) is that the events of student pairs giving the same incorrect answers are random and independent if no collusion occurred. The basic premise of our test is the probability that two students gave the same answer for an MCQ with *Q* = 4 choices is 1/4, assuming that students’ answers are independent. Conversely, this probability would be significantly higher if significant collusion occurred. The rationale for aspect (ii) is that the difference between the probabilities of correctly giving answers to the first and last 20 questions should also be random and on average zero if there was no collusion. On the other hand, given that collusion is more likely to occur during the latter half of the exam, we would expect an increase in the number of correct answers for the last 20 questions.

For aspect (i), we formulated and tested the hypothesis that significant collusion occurred using a set of paired tests^50^. The results of the hypothesis for testing aspect (i) confirm that the corresponding values for each false discovery rate is below the significance of 0.05 (Fig. 2d). Therefore, the empirical evidence does not support that there was an abnormal number of student pairs who consistently gave identical incorrect answers (Supplementary Note 5). To address aspect (ii), we formulated and tested the hypothesis that the difference in means of correctly giving answers to the first and the last 20 questions is zero. Based on the 78 students’ answers to their questions in the exam, we utilized the non-parametric Wilcoxon signed-rank test for paired observations^49^, which yielded a *p* value of 0.3133. Based on the evidence, we cannot reject the hypothesis that the average numbers of correct answers to the first and the last 20 questions are identical. In other words, the difference is not statistically significant between the average numbers of correct answers to the first 20 questions and the last 20 ones.

### Feedback from the post-exam survey

**Fig. 3:**
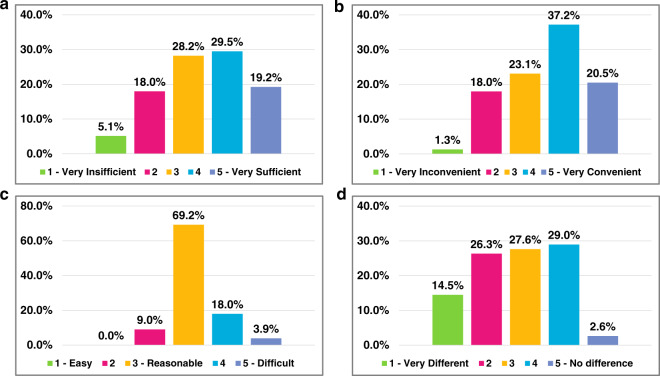
Post-exam online survey results. Bar graph summary in terms of **a** sufficiency of time slot length, **b** convenience of platform interface, **c** easiness of exam questions, and **d** similarity to other online exams respectively (performed on 28 April 2020 for the undergraduate medical imaging course offered at Rensselaer Polytechnic Institute, Troy, New York, USA).

The post-exam survey indicates that the online exam using the DOT platform was well received by a majority of students (Fig. 3). More precisely, 76.9% of students (Fig. 3a) rated the duration for answering questions to be 3 or above out of a 5 point scale ranging from Very Insufficient (1) to Very Sufficient (5), and 80.8% students (Fig. 3b) rated the convenience of using the platform’s interface to be 3 or above on a 5 point scale ranging from Very Inconvenient (1) to Very Convenient (5). The survey also secured feedback concerning the degree of difficulty for the exam questions. Close to 70% of students voted “reasonable”, which is the third choice (Fig. 3c). When excluding the extremes “easy”, or choice number 1, and “difficult”, or choice number 5, 96.1% of students found the questions within the acceptable range (between 2 and 4). The survey finally inquired how similar the final online exam for the students was compared to other online exams they took. The students’ opinions on how familiar the other online exams were with the look-and-feel of our online exam, showing that around 59% of students answered 3 or above out of a 5 point scale ranging from Very Different (1) to No Different (5) (Fig. 3d). The remaining 41% of students indicated that the format of the final exam is different to other exam settings by selecting options (1) and (2).

## Discussions

Although our method is only illustrated with MCQs, our method is actually compatible with most types of questions (except the easy-writing-type tests) since it is the optimized SQs that inhibit the collusion gain. Not to mention that many other types of questions can be easily adapted to the MCQ form. It is also worth noting that our method is compatible with other advanced techniques such as “learning analytics”^29^, which can be integrated into our method for text plagiarism detection in writing-based constructed-response questions.

In the aforementioned post-exam survey, we have received constructive comments from students on how to improve our DOT-format exam design. Specifically, we plan to make the DOT platform more flexible so that questions can have different weights/credits/time-lengths, and both the number of choices and the number of correct choices can be adjusted. Another potential adjustment is to use a soft moving window approach instead of presenting students with one question at a time. Within the soft moving window, a student can work on a small number of questions and amend the answers as needed within the moving time window. It is important to note that such extensions can be similarly analyzed in the discrete optimization framework and do not present any technical difficulty. When prior knowledge of students’ competences is unavailable, an initial phase of an online exam can be devoted to estimate the students’ competence levels. This is then followed by scheduling SQs for the remainder of the exam based on the estimated competence. Finally, the block chain technology^51^ is highly relevant for keeping the database of questions confidential (accessible to faculty only) and managing students’ individual educational credits systematically. These and other improvements can be readily implemented in our optimization framework.

In a recent study^52^, it was shown that COVID-19 could be persistent for years, since thousands of mutations have happened (for example, a SARS-CoV-2 protein had 56% of its genes mutated), explaining many false-negative tests. Over the past several days, the United States experienced a reemerging first or second wave of newly diagnosed cases producing a significantly larger number of infections. Hence, social distancing and equivalent policies due to COVID-19 may remain in place in the near future or even over a longer period of time^53^. A positive response to the pandemic is to let online learning and testing practice enter the mainstream of educational activities or at least it can be assumed to play a significant role while it is being continuously improved. Thanks to the internet and computing technologies, high-quality DOT systems are now feasible solutions in offering comparable exam outcomes that are cost-effective and do not invade students’ privacy.

In conclusion, we have proposed a new type of anti-collusion approach for online exams, which relies on discrete optimization in the permutation space and prior knowledge on students’ competences to suppress collusion behaviors among students. Together with other complementary methods, the general cheating prevention purpose can be achieved. Also, we have reported our DOT platform and its successful application. It has been theoretically, numerically, and experimentally demonstrated that using the DOT technology allows reducing the cheating benefit cost-effectively so that accurate and reliable exams are feasible during social distancing and beyond.

## Methods

### Assumptions on collusion behaviors

The assumptions on collusion behaviors are as follows:

1. Cheating is unidirectional. If two students A and B collaborate on collusion, and A has better competence than B, then only B will copy answers from A which is termed as B cheating from A and A helping B.
2. B can get the answer from A if A has already answered the question before B or they are working on the problem at the same time. Thus, different relative SQs (we denote ‘sequence of questions’ as SQ for short) for A and B will influence the number of questions that B can copy from A.
3. Each student can only cheat from no more than one student (“A helping B” model); Given the limited duration of the time slot for each question and stress involved during an exam, B is expected to rely on typically only one helper A. Put differently, as B requires assistance, he/she is not good at judging which answer is correct when different inputs come from multiple helpers (unless B uses a voting strategy which may or may not make a significant difference to his/her final score). Hence, we consider the “A helping B” model as reasonable in this context.
4. B can help C while cheating from A.
5. B cheating from A does not influence D cheating from A; in other words, one student can help multiple students.
6. An answer based on cheating is not disseminated further to help other students. This assumption can be justified by the argument that given the limited time of an exam and involved stresses, B is unlikely to remember what he/she copied from A and to have the time to provide C with the answer.

### Estimation of students’ competences

The students’ competences are estimated based on their performance in the midterm exam before implementing social distancing. The two classes were taught by different instructors, and have different midterm exams, but they will take the same final at the same time. Thus, their relative performances in the class were treated as their competence score rather than their real scores. The grade distributions of the two classes were first normalized to the distribution with zero mean and unit variance, and then combined together. It is worth mentioning that students who did not participated in the midterm exam were excluded from the normalization procedure, and then put back to the combined profile with 0 (assigning an average performance to estimate their performance). Finally, combined normalized grades were then linearly transformed to the range [0.25, 1] to form the prior knowledge of the competence profile *Y* of the combined set of the students. Note that the range [0.25, 1] is empirically selected. Based on our experience, all questions were covered in the class and a few students got nearly perfect scores while a few totally unprepared students were also seen every semester. In addition, the heuristic colluding matrix *P* relies on the competence differences rather than the competence values, hence, the linear transformation of the competence range will only impose a constant scaling factor on the average collusion gain *g* base on equation (1), which will not influence the optimization result of the SQ assignments.

### Construction of the colluding matrix

To perform the optimization, we heuristically construct a colluding matrix *P* depicting the probability of every student cheating from another student. Following the notation in the main text, reasonable assumptions about colluding mechanisms are made as follows: (1) The probability of student *i* actively cheating is related to his/her competence *y*_*i*_; Student 1 tends not to cheat since he/she could obtain no gain (risk greater than benefit), while student *N* will try all means to cheat since he/she will always gain (benefit greater than risk). (2) The probability of collusion happens between two students A and B is related to the difference of *y*_A_ and *y*_B_. Student *i* will have the strongest willingness to cheat from student 1, but the least willingness to cheat from student *j* if *y*_*i*_ = *y*_*j*_ since he/she cannot trust *j* more than himself/herself, and he/she will never cheat from *j* if *y*_*i*_ > *y*_*j*_.

Based on the assumptions above, the colluding matrix *P* is heuristically constructed as follows:

3 $${p}_{j,i}=\left\{\begin{array}{ll}0,&{y}_{j}\le {y}_{i}\\ \frac{{y}_{j}-{y}_{i}}{\mathop{\sum }\nolimits_{k = 1}^{{n}_{f}(i)}({y}_{k}-{y}_{i})}(1-{p}_{i,i}),&{y}_{j}\;>\;{y}_{i}\end{array}\right.$$

4 $${p}_{i,i}={\left[1-\frac{\mathop{\sum }\nolimits_{k = 1}^{{n}_{f}(i)}({y}_{k}-{y}_{i})}{\mathop{\sum }\nolimits_{k = 1}^{N}({y}_{k}-{y}_{N})}\right]}^{\eta }$$

where *n*_*f*_(*i*) is defined as the number of elements in *Y* that are greater than *y*_*i*_, and *η* is a non-negative constant which can be used to adjust students’ willingness to cheat. Larger *η* will increase the colluding probability, and students are supposed to always commit active cheating if *η* = *∞* (all optimizations were conducted with this setting). Equations (3) and (4) define the probabilities of the cheating and non-cheating states of student *i* respectively, and in the cheating state, the possibility of student *i* will cheat from student *j* is proportional to their competence difference *y*_*j*_ − *y*_*i*_ normalized by the sum of competence differences in all possible cases.

Without loss of generality, we further assume that students have different competences (*y*_1_ > *y*_2_ > ⋯ > *y*_*N*_), due to the fact that adding tiny differences to two equal *y* has a negligible effect on the result of *g* and simplify the expression of *n*_*f*_(*i*) to be of the form

5 $${n}_{f}(i)=i-1$$

Hence, *p*_*j*,*i*_ can be written more explicitly as follows:

6 $${p}_{j,i}=\left\{\begin{array}{ll}0,&j<{i}\\ (1-{p}_{i,i})({y}_{j}-{y}_{i})/(\mathop{\sum }\nolimits_{k = 1}^{i}{y}_{k}-i{y}_{i}),&j>{i}\\ {\left[1-\mathop{\sum }\nolimits_{k = 1}^{i}({y}_{k}-{y}_{i})/\mathop{\sum }\nolimits_{k = 1}^{N}({y}_{k}-{y}_{N})\right]}^{\eta },&j=i\end{array}\right.$$

Note that the heuristic colluding matrix *P* represents a practically reasonable start for optimization. We construct *P* to place a larger weight on the collusion between students with a larger competence difference than that with a small competence difference, which helps limit the collusion gain in the worst-case scenario. Since mismatches exist very likely between the model and the practice, any optimization result needs to be subjected to a worst-case analysis.

### Analysis of worst-case metrics

Similar to the average case analysis and worst-case analysis in computer science, we may want to revisit our optimized results in a worst-case study since mismatch is very likely to exist between the model and the practice. Hence, another two important metrics are introduced to assess the optimization results from the risk control angle, i.e., the worst-case average collusion gain *g*_W_ defined as the average collusion gain in the situation where all students manage to achieve their maximum possible collusion gain (the maximum possible collusion gain of the student *i* is achieved by setting the probability of *i* cheats with the student *j* to 1, from whom *i* will obtain the maximum gain among other choices of *i*),

7 $${g}_{\mathrm W}(A)=\frac{1}{N{M}_{1}}\,\text{sum}\,\left\{\right.\mathop{\max }\limits_{j\in [N]}\{Z(A)\circ D\}\left\}\right.$$

and the maximum individual collusion gain *g*_MI_ which is the maximum of the maximum possible collusion gains over all students,

8 $${g}_{\mathrm {MI}}(\mathrm A)=\frac{1}{{M}_{1}}\mathop{\max }\limits_{i,j\in [N]}\{Z(A)\circ D\}.$$

*g*_W_ can be used to assess the performance of the optimized results under the worst situation and can be treated as a reliable upper limit estimation of the collusion gain under the given competence profile *Y* since the calculation of *g*_W_ does not involve the colluding matrix. *g*_MI_ is a metric can be used to estimate the fairness of the exam from the aspect of the maximum collusion gain any student can achieve. If the collusion gain calculated in the worst situation for the output assignment is not acceptable, the result should be used with caution or just change the initialization and generate more solutions. Overall notations and metrics of the model are summarized in Supplementary Tables 1 and 2.

### Cyclic greedy searching

In principle the optimal assignment to achieve the minimized collusion gain should be searched from the set of all possible assignments whose size is *n*^*N*^ and *n* is the size of the pool of SQs *P*_SQ_. Practically, an optimal solution will be computationally infeasible (seemingly NP-hard) if there are many students and/or many questions in the exam, hence we propose the following efficient algorithm. We first narrow the searching pool of SQs to the sequences generated by circular shifting (let us denote the set as *P*_CS_) from *P*_SQ_ following the heuristic that *P*_CS_ is a good representative subspace of *P*_SQ_. *P*_CS_ contains all possible *z* values achieved by any two sequences from *P*_SQ_, and if we randomly choose two sequences from the two space, the expected *z* value of two sequences from *P*_CS_ is even smaller than that from *P*_SQ_ (see Supplementary Note 7 for the proof). Then, we choose to use a greedy-searching algorithm from a randomly initialized assignment or the assignment generated with the result of GAS, and repeat the searching process for multiple times until the loss does not decrease. Through this greedy searching, satisfactory results can be easily obtained in polynomial time.

Specifically, we can perform Greedy Searching from a Cyclic pool (Cyclic Greedy Searching, CGS). The concept behind CGS is to iterate with respect to each and every student, and replace his/her current sequence of questions with one from *P*_CS_ if the updated assignment achieves a smaller average collusion gain. Several cycles of greedy-searching are needed to fulfill a complete search, and the output assignment from the last cycle will be treated as the initialization for the next cycle during the iteration. We use the result from GAS as our preferred initialization, and other initialization is also suggested to be adopted and find the best one among the results to improve the solution (see Algorithm 2 in Supplementary Note 2 for pseudocodes and implementation details).

### Min–max greedy matching

Instead of searching in the cyclic pool *P*_CS_, we can search from the entire permutation pool *P*_SQ_ to minimize the collusion gain. Due to hardness of searching in a huge permutation space when the problem scale is large, we adapted the min–max greedy matching algorithm (MMM) to work in polynomial time. A natural approach is to start with an initial random assignment and improve it greedily by picking up one student at a time according to a certain order, and refining his/her SQ so that the total gain is minimized from the set of all possible *M*_1_-permutations of *M*_2_. We propose MMM to greedily improve an assignment, and show that computing a sequence to replace a single student’s sequence in an assignment that minimizes the total gain can be done in polynomial time by performing a minimum weight maximum matching (see Algorithm 3 in Supplementary Note 7 for implementation details). For convenience, we first introduce some notations. Given any *s* ∈ *P*_*SQ*_:

1. For each *j* ∈ [*M*_2_], we define *s*(*j*) = *l* if *j* appears in the *l*th position in *s*, and *s*(*j*) = 0 otherwise.
2. For each *j* ∈ [*M*_2_], *α*(*s*, *j*) = 1 if *s*(*j*) ≥ 1, and *α*(*s*, *j*) = 0 otherwise, to indicate whether question *j* is on sequence *s*.
3. For each *j* ∈ [*M*_2_], each *l*≤*M*_1_, *β*(*s*, *j*, *l*) = 1 if *s*(*j*) ≥ 1, *s*(*j*) ≤ *l*, and *β*(*s*, *j*, *l*) = 0 otherwise, to indicate whether question *j* appears at or before position *l* on sequence *s.*
4. For each *j* ∈ [*M*_2_], each *l*≤*M*_1_, *γ*(*s*, *j*, *l*) = 1 if *s*(*j*) ≥ *l*, and *γ*(*s*, *j*, *l*) = 0 otherwise, to indicate whether question *j* appears at or after position *l* on sequence *s*.
5. For any $s,s^{\prime} \in {P}_{SQ}$, and any *j* ∈ [*M*_2_], $\delta (s,s^{\prime} ,j)=1$ if *s*(*j*) > 1, $s^{\prime} (j)>1$, and $s^{\prime} (j)\le s(j)$, and $\delta (s,s^{\prime} ,j)=0$ otherwise to indicate whether a student assigned *s* can cheat on question *j* from a student assigned $s^{\prime}$.

Given an instance ([*N*], [*M*_2_], [*M*_1_], *Y*), MMM is initialized with an assignment *A*, and proceeds to greedily improve *A* in *N* rounds, one student at a time, as follows: In each round *i* ≤ *N*, student *i* is selected, and *a*_*i*_ is greedily replaced by the sequence s^*^ that minimizes total gain, or simply restated, provides the largest drop in the average gain from *A*. Formally,

9 $${s}^{* }=\arg \mathop{\min }\limits_{s\in {P}_{SQ}}g((s,{a}_{-i}))$$

10 $$=\arg \mathop{\min }\limits_{s\in {P}_{SQ}}g((s,{a}_{-i}))-g(A)$$

where (*s*, *a*_−*i*_) denotes the assignment where student *i*’s sequence *a*_*i*_ is replaced with *s*. Note that for any *s* ∈ *P*_SQ_, the difference in the average gain between (*s*, *a*_−*i*_) and *A* is the sum of the differences in the gain from each question *j* that appears in the sequence *s*, as shown in Eq. (11).

11 $$\begin{array}{lll}g((s,{a}_{-i}))-g(A)&=&\frac{1}{N}\sum\limits_{j\in s}\left[\sum\limits_{k\,\le\, i}{p}_{k,i}[{y}_{k}\beta({a}_{k},j,s(j))+{y}_{i}(1-\beta ({a}_{k},j,s(j)))] -[{y}_{k}\delta ({a}_{i},{a}_{k},j)+{y}_{i}(1-\delta ({a}_{i},{a}_{k},j))]\right.\\ &&\left.+\sum\limits_{h\,{>}\,i}{p}_{i,h}[{y}_{i}\gamma ({a}_{i},j,s(j))+{y}_{h}(1-\gamma ({a}_{i},j,s(j)))] [{y}_{i}\delta ({a}_{h},{a}_{i},j)+{y}_{h}(1-\delta ({a}_{h},{a}_{i},j))]\right]\end{array}$$

We compute ${s}^{* }=\arg \mathop{\min }\limits_{s\in {P}_{SQ}}g((s,{a}_{-i}))$ by solving the following minimum weight maximum matching problem to match questions to positions in a sequence. We define a weighted, complete, bipartite graph *G* = ([*M*_1_] ∪ [*M*_2_], *E*) with a node for each of *M*_1_ positions, and a node for each of *M*_2_ questions. For each pair of a position *l* ∈ [*M*_1_] and question *j* ∈ [*M*_2_], we set the weight of the edge to be the difference in the gain from question *j* when it appears in position *l* and the gain from question *j* as it appears in *a*_*i*_, w.r.t. the sequences *a*_−*i*_ of all of the other students. It is easy to see that solving this minimum weight maximum matching problem assigns student *i* with a desired sequence of *M*_1_ questions ${s}^{* }=\arg \mathop{\min }\limits_{s\in {P}_{SQ}}g((s,{a}_{-i}))-g(A)=\arg \mathop{\min }\limits_{s\in {P}_{SQ}}g((s,{a}_{-i}))$.

Then, we extended MMM into the MMM-CGS algorithm as a natural extension of MMM and CGS by setting the initial assignment to the output of CGS (modifying line 2 in Algorithm 3 in the Supplementary Note 2) and improving it greedily in the same manner as MMM. This ensures that we will only improve solutions from the CGS (at least no harm), which implies a room for potential improvement of our heuristic optimization method CGS.

### Integer linear programming (ILP)

For the optimal performance, we adapted this setting into an integer linear programming problem to find an optimal assignment in the permutation space but at an exponential computational cost, as shown in Algorithm 4 in Supplementary Note 2.

We begin by showing correctness of Algorithm 4, and that it computes a valid solution. Consider an arbitrary instance *I* = ([*N*], *M*_2_, *M*_1_, *Y*), and let *A* be the assignment returned by Algorithm 4 when applied on this instance *I*. It is easy to see that for any student *i* ∈ [*N*], (ii) for any question *j* ∈ [*M*_2_], there is at most one value of *l* ∈ [*M*_1_], such that *s*_*i*,*j*_ = *l*, otherwise the constraint ${\sum }_{j\in [{M}_{1}]}{m}_{i,j,l}=1$ is violated, and (ii) for any position *l* ∈ [*M*_1_], there is exactly one question *j* ∈ [*M*_2_] such that *s*_*i*,*j*_ = *l*, otherwise, together with (i), the constraint ${\sum }_{j\in [{M}_{1}]}{s}_{i,j}={\sum }_{l\in [{M}_{2}]}l$ is violated. It is easy to see by the construction of Algorithm 4, that every student is assigned *M*_1_ questions in *A* in a valid sequence.

It is easy to verify that the objective of the ILP formulation in Algorithm 4 is the score of the assignment indicated by the variables *s*_*i*,*j*_, by checking that for each pair of students *i*, *k* ∈ [*N*], and for each question *j* ∈ [*M*_2_], the variables *c*_*i*,*k*,*j*_ correctly indicate whether *i* can copy from *k* on question *j* under the assignment indicated by the variables *s*_*i*,*j*_ and *s*_*k*,*j*_.

To prove completeness, it is sufficient to verify that every possible assignment is a feasible solution to the ILP in Algorithm 4. It is easy to check that for every valid assignment *A*, there is a way to assign values first to variables *s*_*i*,*j*_ corresponding to the sequences in *A*, and subsequently to the rest of the variables in the ILP formulation in a manner that does not violate any of the constraints.

### Practical guideline

The GAS itself is usually not optimal, but using its result as the initialization of the greedy algorithms can guarantee the theoretical bound of the average collusion gain of the searching results. In our simulation, the performance of our fast heuristic search algorithm CGS was close to the results optimized using the two sophisticated algorithms MMM and ILP (Supplementary Note 3). Note that our CGS method does not guarantee convergence on the optimum, and is theoretically different from the competitive sophisticated algorithms. Especially, the ILP algorithm finds the global optimum but requires exponentially more computational resources. To design online exams of small scales, we generally prefer using ILP as appropriate. For online exams of large scales, we generally prefer using the MMM algorithm that is of polynomial complexity to find at least a local minimum, initialized by the output of our GAS and CGS methods.

### Data collection and ethics oversight

We developed a DOT platform (a web application, all MCQs, detailed in Supplementary Note 4 and 6) to perform the online test. The post-exam survey was performed through SurveyMonkey. We have complied with all relevant ethical regulations. The RPI Institutional Review Board approved the study protocol. The informed consent was obtained from all participants in the study.

### Reporting summary

Further information on research design is available in the Nature Research Reporting Summary linked to this article.

## Supplementary information

Supplementary Information for Optimized Collusion Prevention for Online Exams during Social Distancing

Reporting Summary

## Data Availability

We declare that all data that support the findings of this study are already available within the manuscript and the supplementary information. Raw data of the tests will be made available upon request and after going through an Institutional Review Board procedure at RPI.

## Code availability

The source codes and simulation data for this study are publicly available on Github (https://github.com/edward1001001/Anticheating_in_DOT).
