# Supplementary material for: Optimized collusion prevention for online exams during social distancing
Source: NPJ Sci Learn. 2021 Mar 1;6:5. doi: 10.1038/s41539-020-00083-3 (PMC7921656; doi:10.1038/s41539-020-00083-3)
Supplement: Supplementary file 1 — Supplementary Information for Optimized Collusion Prevention for Online Exams during Social Distancing [file 41539_2020_83_MOESM1_ESM.pdf]

Supplementary Information for Optimized Collusion Prevention for Online Exams during Social Distancing

Mengzhou Li,[1] Lei Luo,[2] Sujoy Sikdar,[3] Navid Nizam,[1] Shan Gao,[1] Hongming Shan,[1] Melanie Kruger,[4] Uwe Kruger,[1] Hisham Mohamed,[1] Lirong Xia,[2] and Ge Wang[1*]

[1]Department of Biomedical Engineering, Rensselaer Polytechnic Institute, Troy, NY 12180, USA

[2]Department of Computer Science, Rensselaer Polytechnic Institute, Troy, NY 12180, USA

[3]Department of Computer Science and Engineering, Washington University in St. Louis, St. Louis, MO 63130, USA

[4]Department of Mechanical, Aerospace and Nuclear Engineering, Rensselaer Polytechnic Institute, Troy, NY 12180, USA

[*]To whom correspondence should be addressed; E-mail: wangg6@rpi.edu.

Supplementary Figures

Supplementary Figure 1 Supplementary illustration of the GAS. 1

Supplementary Tables

Supplementary Table 1 Summation of Notations 2
Supplementary Table 2 Summation of Metrics. 3

Supplementary Notes

1 Proof of Collusion Control Theorem 4

2 Anti-Collusion Algorithms 5

3 Performance Comparison of the Algorithms 10

4 Final Exam Design 14

5 Statistical Testing: Did Significant Collusion Occur 18

6 DOT Platform 20

7 Random Sampling 23

Supplementary Figure 1

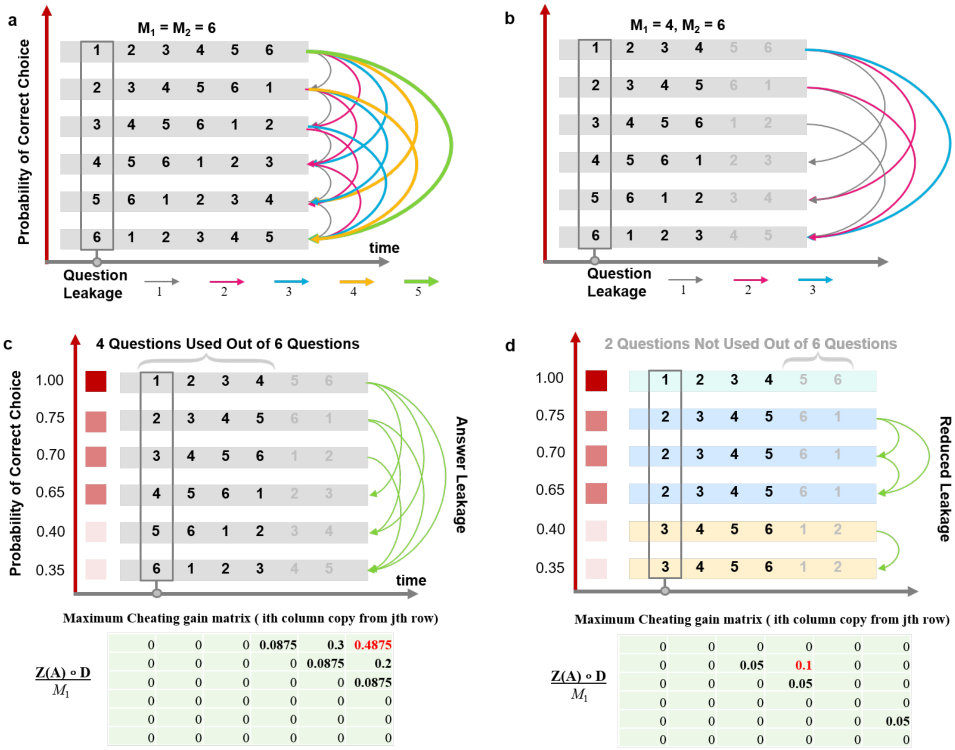

Supplementary Figure 1: Supplementary illustration of the grouping-based anti-collusion scheme (GAS). Circular shifting demos with **a**, $M_2 = M_1 = 6$, and **b**, $M_2 = 6$, $M_1 = 4$ demonstrate that using consecutive sequences and reducing the test length M_1 help reduce the maximum question leakage; A simple example of 4 questions from the pool of 6 questions properly assigned to 6 students for suppression of the collusion gain via grouping: **c**, primitive assignment of four questions (in black) to six students via circular shifting, yielding a maximum individual collusion gain 48.75% and an average collusion gain in the worst case $\sim 14.6\%$, and **d**, an assignment follows the general grouping-based anti-collusion scheme, reducing the maximum individual collusion gain to 10% and the average collusion gain in the worst case $\sim 3\%$. Note that in **d**, the students are grouped by similarity in their competence levels to bound the maximum collusion gain.

Supplementary Table 1

Supplementary Table 1: Summation of Notations

Symbol	Definition
N	The number of *students*, $[N] = \{1, 2, \cdots, N\}$ means the student set.
M_2	The number of *questions* in the MCQ bank, $[M_2]$ means the total question set.
M_1	The number of questions to be asked each student.
Q	The number of *choices* for each MCQ and only one of them is correct.
Y	The *competence profile* of students, $y_i \in [1/Q, 1]$ $(i \in [N])$ represents the probability of student i correctly answers a question. y_i in Y is ranked in a descending order.
P	The *colluding matrix*, $p_{j,i}$ $(i, j \in [N])$ depicts the probability for student i to actively cheat from student j if $i \neq j$ (The first index indicates the source of the answers while the second indicates the destination of the information). $p_{j,i} = 0$ if $j > i$ due to the assumption (1), and $p_{i,i} = 1 - \sum_{j=1}^{i-1} p_{j,i}$.
A	The *assignment*, $A = \{a_i \in P_{SQ} \| i = 1, 2, \cdots, N\}$, composed of a set of SQs from P_{SQ} and depicting the mapping from students $[N]$ to the permutation pool of SQs P_{SQ}, and a_i represents the SQ assigned to student i. s_i mapping from the students to P_{SQ}
P_{SQ}	All possible M_1-length SQs formed by the permutation of M_2 MCQs, which is also referred as the *permutation pool*.
n	The size of the permutation pool P_{SQ}, $n = M_2!/(M_2 - M_1)!$.
$q_i(A)$	The expected score of student $i \in [N]$ with collusion in the exam with assignment A, which can also be referred as the *cheating score* for short. Noted that $q_i(A)$ is also related to how P is defined.
q_i^*	The expected honest score of student $i \in [N]$ without collusion (irrelevant to sequence assignment A), which can also be referred as the *honest score* for short.
Z	The *positional matrix*, and $z_{j,i}$ $(i, j \in [N])$ depicts the number of questions that student i can cheat from student j if $j \neq i$, and the special case $z_{i,i}$ is defined as M_1. Noted that Z is directly calculated from the assignment A and irrelevant to P or Y.
D	The *competence difference matrix* D defined as $(d_{j,i})_{i,j \in [N]}$ and $d_{j,i} = \max(y_j - y_i, 0)$ for the easy of expression of other variables.
g_i	The *expected collusion gain* for student i under assignment A is defined as the difference between his/her cheating score and honest score, calculated by $q_i(A) - q_i^*$.

Supplementary Table 2

Supplementary Table 2: Summation of Metrics.

Metric	Definition
g	The *average collusion gain* g is defined as the sum of expected collusion gains over all students $[N]$, which is also the main metric that our optimizations tend to minimize. $g(A) = \frac{1}{N}\sum_{i=1}^{N} g_i(A)$.
g_W	The *worst case average collusion gain* g_W is defined as the average collusion gain g in the situation where all students manage to achieve their maximum possible collusion gain (the maximum possible collusion gain of the student i is achieved by setting the probability of i cheats with the student j to 1, from whom i will obtain the maximum gain among other choices of i); $g_W(A) = \frac{1}{NM_1}\text{sum}\big\{\max_{j\in[N]}\{Z(A) \circ D\}\big\}$.
g_{MI}	The *maximum individual collusion gain* is the maximum of the maximum possible collusion gains for all students ($\max_i[\max_P(g_i)]$). $g_{MI}(A) = \frac{1}{M_1}\max_{i,j\in[N]}\{Z(A) \circ D\}$, where $\circ$ stands for Hadamard multiplication (element-wise).

Note: g_W and g_{MI} are irrelevant to P, and can be used to revisit the optimized results for the worst case analysis. g_W can be treated as a reliable upper limit estimation of the collusion gain under the given competence profile Y. g_{MI} can be used to estimate the fairness of the exam from the aspect of the maximum collusion gain any student can achieve.

1 Proof of Collusion Control Theorem

Theorem 1. *Given sequences of M_1 questions from the bank of M_2 MCQs and with only one correct choice out of Q choices for each question, the maximum individual collusion gain is no more than $(1-1/Q)/(M_2-M_1+1)$ using the grouping-based anti-collusion scheme.*

Proof. For any two same length question sequences s_1 and s_2, let $F_z(s_1, s_2)$ stand for the number of questions that can be copied from s_1 to s_2. Let us denote the M_2 questions as $\{1, 2, 3, \ldots, M_2\}$.

Following the grouping-based anti-collusion scheme, (1) by circular shifting, we can easily create $M_2 - M_1 + 1$ sequences: $s_1 = [1, 2, 3, \ldots, M_1]$, $s_2 = [2, 3, \ldots, M_1, M_1 + 1]$, ..., $s_{M_2-M_1+1} = [M_2 - M_1 + 1, M_2 - M_1 + 2, \ldots, M_2]$. It is easy to check for any pair of s_i and s_j out of them, we have $F_z(s_i, s_j) = 0$ if $i < j$. (2) Let us divide the maximum possible student competence score range $[1/Q, 1]$ into $M_2 - M_1 + 1$ intervals, and the length of each interval is $(1-1/Q)/(M_2-M_1+1)$, as shown in Supplementary Figure 2. Then, we can group the students whose competences are in the same interval, and obtain $M_2 - M_1 + 1$ groups. We assign the sequences $s_1, s_2, \ldots, s_{M_2-M_1+1}$ to the groups ranked in the descend order of their competences. By doing so, we have achieved two goals: first, there is no collusion gain between groups ensured by (1); second, the individual maximum gain inside a group cannot be greater than $(1 - 1/Q)/(M_2 - M_1 + 1)$ which is the interval length, ensured by (2), and regardless of how many students are in the group. Hence, we have proved the theorem. ■

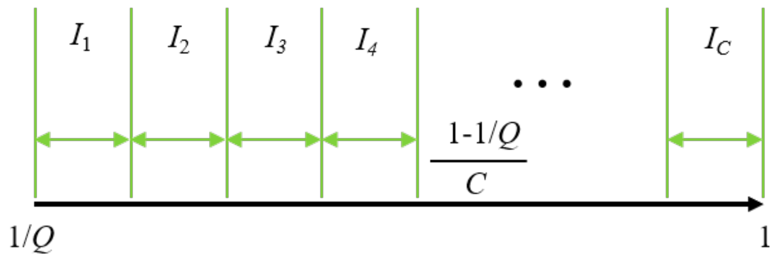

Supplementary Figure 2: Illustration of the collusion control theorem to control the maximum individual collusion gain to be below any desired level. By dividing the competence range into C intervals and grouping students into these intervals can controls the maximum individual gain below the length of the interval $(1 - 1/Q)/C$, where $C = M_2 - M_1 + 1$.

2 Anti-Collusion Algorithms

2.1 Algorithm 1: Grouping-based Anti-collusion Scheme (GAS)

For general exam designs with flexible M_2 and M_1, we can just replace the $M_2 - M_1 + 1$ with the predefined C in line 4 of the Algorithm 1.

Algorithm 1 GAS

```
1: Input: A DOT instance ([N], [M_2], M_1, Y).
2: Generate P_CS = {s_1, s_2, ..., s_{M_2}} from [M_2] by left circular shifting
3: Sort Y in the descending order
4: Partition [min(Y), max(Y)] into M_2 - M_1 + 1 intervals I_1, I_2, ..., I_{M_2-M_1+1} with equal
   length.
5: Initialize the index for interval t = 1.
6: for i = 1, ..., N, do
7:   if y_i ∈ I_t then
8:     a_i ← s_t          ▷ Students inside one interval receive the same sequence
9:   else
10:    t = t + 1;
11:    a_i ← s_t
12: return A.
```

2.2 Algorithm 2: Cyclic Greedy Searching (CGS)

Building on Algorithm 1, we propose CGS (Algorithm 2) as an extension to search P_{CS} to greedily improve upon the assignment computed by Algorithm 1. Algorithm 2 proceeds in two phases as follows: In Phase 1, we use the result A_0 from Algorithm 1 as our preferred initialization, and together with other reasonable/random initializations we will find the best one among the respectively optimized results. In Phase 2, in each of N rounds, a student is selected based on the competence order from high to low, and a sequence which minimizes the average collusion gain is selected from P_{CS} to be assigned to the student (the assignment is updated only if the update reduces current average collusion gain to ensure convergence). For any assignment A, we will use (s, a_{-i}) to denote the assignment where student i's sequence a_i is replaced with a sequence $s \in P_{CS}$. The steps in Phase 2 will be repeated for a maximum of N_{rep} times or until a local minima is reached.

Algorithm 2 CGS

Input: A DOT instance $([N], [M_2], M_1, Y, P, A_0)$.
Generate $P_{CS} = \{s_1, s_2, \ldots, s_{M_2}\}$ from $[M_2]$ by left circular shifting
$A \leftarrow A_0$. ▷ Initialization A_0
$N_{rep} = 30$. ▷ Maximum repetition time
$tA = A_0$.
for $iter = 1, \ldots, N_{rep}$, **do**
  for $i = 1, \ldots, N$, **do** ▷ Greedy improvements
    for $j = 1, \ldots, M_2$, **do**
      if $g((s_j, a_{-i}), P) < g(A, P)$ **then**
        $a_i \leftarrow s_j$
  if $tA == A$ **then**
    break ▷ Assignment does not change, and stop
  else
    $tA = A$
return A.

2.3 Algorithm 3: Min-Max Greedy Matching (MMM)

Our next algorithms remove the restriction on the search space to the cyclic pool P_{CS}, and search for assignments in the pool of all possible question sequences P_{SQ}. The pseudo code of Algorithm 3 is presented below, and some notations are copied from the main text for clarity. Given any $s \in P_{SQ}$:

1. For each $j \in [M_2]$, we define $s(j) = l$ if j appears in the l-th position in s, and $s(j) = 0$ otherwise;
2. For each $j \in [M_2]$, $\alpha(s, j) = 1$ if $s(j) \geq 1$, and $\alpha(s, j) = 0$ otherwise, to indicate whether question j is on sequence s;
3. For each $j \in [M_2]$, each $l \leq M_1$, $\beta(s, j, l) = 1$ if $s(j) \geq 1$, $s(j) \leq l$, and $\beta(s, j, l) = 0$ otherwise, to indicate whether question j appears at or before position l on sequence s;
4. For each $j \in [M_2]$, each $l \leq M_1$, $\gamma(s, j, l) = 1$ if $s(j) \geq l$, and $\gamma(s, j, l) = 0$ otherwise, to indicate whether question j appears at or after position l on sequence s;
5. For any $s, s\prime \in P_{SQ}$, and any $j \in [M_2]$, $\delta(s, s\prime, j) = 1$ if $s(j) > 1$, $s\prime(j) > 1$, and $s\prime(j) \leq s(j)$, and $\delta(s, s\prime, j) = 0$ otherwise to indicate whether a student assigned s can cheat on question j from a student assigned $s\prime$.

Algorithm 3 MMM

Input: A DOT instance $([N], [M_2], [M_1], Y)$**.**
$A \leftarrow$ a random assignment.
for $i \leq N$, **do**
  $G \leftarrow ([M_1] \cup [M_2], E = \{(l, j) : l \leq M_1, j \leq M_2\})$. ▷ bipartite
  for $l \leq M_1, j \leq M_2$, **do** ▷ Edge weights are the marginal gain over A by placing j as i's l-th question.
    $w_{(l,j)} \leftarrow 0$.
    for $k \leq i$, **do**
      $w_{(l,j)} \leftarrow w_{(l,j)} + p_{k,i}\big[[y_k\beta(a_k, j, l) + y_i(1 - \beta(a_k, j, l))] - [y_k\delta(a_i, a_k, j) + y_i(1 - \delta(a_i, a_k, j))]\big]$
    for $h > i$, **do**
      $w_{(l,j)} \leftarrow w_{(l,j)} + \sum_{h>i} p_{i,h}\big[[y_i\gamma(a_i, j, l) + y_h(1 - \gamma(a_i, j, l))] - [y_i\delta(a_h, a_i, j) + y_i(1 - \delta(a_h, a_i, j))]\big]$
  $Matching \leftarrow$ minimum weight maximum matching on G.
  For each $(l, j) \in Matching$, $a_{i,l} \leftarrow j$.
return A.

2.4 Algorithm 4: Integer Linear Programming (ILP)

We cast the assignment optimization problem into an integer linear programming problem to find a globally optimal assignment in the permutation space, as shown below in Algorithm 4.

Algorithm 4 ILP

Input: A DOT instance $([N], [M_2], M_1, Y)$**.**

Solve the ILP in Supplementary Figure 3 below and set assignment A as follows: for each $i \in [N], j \in [M_2]$, such that $s_{i,j} > 0$, $a_i(j) \leftarrow s_{i,j}$.

return A.

$\min_{s} \sum_{i\in[N],j\in[M_2]} q_{i,j},$		objective
Variables		
$q_{i,j} \in [0,1],$	$\forall i \in [N], j \in [M_2]$	expected scores
$s_{i,j} \in \{0,1,\dots,M_1\},$	$\forall i \in [N], j \in [M_2]$	rank of question
$x_{i,j} \in \{0,1\},$	$\forall i \in [N], j \in [M_2]$	indicate assignment
$e_{i,j,l} \in \{0,1\},$	$\forall i \in [N], j \in [M_2], l \in [M_1]$	indicate $s_{i,j} \geq l$
$f_{i,j,l} \in \{0,1\},$	$\forall i \in [N], j \in [M_2], l \in [M_1]$	indicate $s_{i,j} \leq l$
$m_{i,j,l} \in \{0,1\},$	$\forall i \in [N], j \in [M_2], l \in [M_1]$	indicate $s_{i,j} = l$
$c_{i,k,j} \in \{0,1\},$	$\forall i \in [N], k \in [N], j \in [M_2]$	indicate i can copy k on j
$u_{i,k,j} \in \{0,1\},$	$\forall i \in [N], k \in [N], j \in [M_2]$	indicate both i, k assigned j
$v_{i,k,j} \in \{0,1\},$	$\forall i \in [N], k \in [N], j \in [M_2]$	indicate k sees j before i
Constraints		
$\sum_{j\in[M_2]} s_{i,j} = \sum_{l\in[M_1]} l,$	$\forall i \in [N]$	feasible assignment
$x_{i,j} \geq \frac{s_{i,j}}{M_2},$	$\forall i \in [N], j \in [M_2]$	feasible assignment
$x_{i,j} \leq s_{i,j},$	$\forall i \in [N], j \in [M_2]$	feasible assignment
$e_{i,j,l} \geq \frac{1+s_{i,j}-l}{M_2+1},$	$\forall i \in [N], j \in [M_2], l \in [M_1]$	feasible assignment
$e_{i,j,l} \leq \frac{M_2+s_{i,j}-l}{M_2},$	$\forall i \in [N], j \in [M_2], l \in [M_1]$	feasible assignment
$f_{i,j,l} \geq \frac{1+l-s_{i,j}}{M_2+1},$	$\forall i \in [N], j \in [M_2], l \in [M_1]$	feasible assignment
$f_{i,j,l} \leq \frac{M_2+l-s_{i,j}}{M_2},$	$\forall i \in [N], j \in [M_2], l \in [M_1]$	feasible assignment
$m_{i,j,l} \leq e_{i,j,l},$	$\forall i \in [N], j \in [M_2], l \in [M_1]$	feasible assignment
$m_{i,j,l} \leq f_{i,j,l},$	$\forall i \in [N], j \in [M_2], l \in [M_1]$	feasible assignment
$m_{i,j,l} \geq e_{i,j,l} + f_{i,j,l}$ - 1,	$\forall i \in [N], j \in [M_2], l \in [M_1]$	feasible assignment
$\sum_{j\in[M_2]} m_{i,j,l} = 1,$	$\forall i \in [N], l \in [M_1]$	feasible assignment
$u_{i,k,j} \leq x_{i,j},$	$\forall i \in [N], k \in [N], j \in [M_2]$	both assigned j
$u_{i,k,j} \leq x_{k,j},$	$\forall i \in [N], k \in [N], j \in [M_2]$	both assigned j
$u_{i,k,j} \geq x_{i,j} + x_{k,j}$ - 1,	$\forall i \in [N], k \in [N], j \in [M_2]$	both assigned j
$v_{i,k,j} \geq \frac{1+s_{i,j}-s_{k,j}}{M_2+1},$	$\forall i \in [N], k \in [N], j \in [M_2]$	k has seen j
$v_{i,k,j} \leq \frac{M_2+s_{i,j}-s_{k,j}}{M_2},$	$\forall i \in [N], k \in [N], j \in [M_2]$	k has seen j
$c_{i,k,j} \leq u_{i,k,j},$	$\forall i \in [N], k \in [N], j \in [M_2]$	i can copy k on j
$c_{i,k,j} \leq v_{i,k,j},$	$\forall i \in [N], k \in [N], j \in [M_2]$	i can copy k on j
$c_{i,k,j} \geq u_{i,k,j} + v_{i,k,j}$ - 1,	$\forall i \in [N], k \in [N], j \in [M_2]$	i can copy k on j
$q_{i,j} = \sum_{k\in[N]} y_k p_{k,i} c_{i,k,j} +$, $y_i(1 - \sum_{k\in[N]} p_{k,i} c_{i,k,j})$	$\forall i \in [N], j \in [M_2]$	expected score

Supplementary Figure 3: ILP to compute an assignment with minimum gain.

3 Performance Comparison of the Algorithms

We evaluate the performance of the proposed algorithms in the previous section on multiple synthetic datasets with different settings corresponding to the choice of values for N, M_2, and M_1, over 100 instances for each setting. For each setting, we generate the competences of N students i.i.d. uniformly at random from $[0.25, 1)$, and generate the colluding probabilities for each student $i \in \{2, \ldots, N\}$ to cheat from the students $k < i$ ($i - 1$ in total) from the ($i - 1$)-variate $Dirichlet$ distribution with a concentration parameter of $\alpha = 10$, meaning that $\sum_{k<i} p_{k,i} = 1$ and $p_{1,1} = 1$ in our experiments. In addition to random assignments and assignments obtained directly by circular shifting, we consider four algorithms: **CGS** (Algorithm 2), **MMM** (Algorithm 3), **MMM-CGS**, and **ILP** (Algorithm 4, which computes an optimal assignment with minimized gain). CGS was first initialized with the assignment generated from GAS which provides a proven upper bound of the collusion gain, and then was randomly initialized from the cyclic pool P_{CS} for 9 times, and the best result was selected for comparisons and used as the initialization of MMM-CGS. Experiments were performed on a computer equipped with a AMD Ryzen 7 2700X processor running at 4.0GHz and 16GB of system memory. Due to practical considerations of running time and system memory, we evaluate against the ILP on instances with at most $N = 10$, $M_2 = 5$, and $M_1 = 3$. We evaluate the relative performance of our greedy heuristic algorithms on larger instances with $N = 100$, $M_2 = 30$, and different values of $M_1 \in \{10, 20\}$.

Supplementary Figure 4 presents our experimental results. For each of the four algorithms and additionally random and cyclical assignments (Y-axis), Supplementary Figure 4 shows the gain over the honest score computed using Equation 7 (from the main text) (X-axis) over 100 instances, with a box representing the upper and lower quantiles, an orange line within the box representing the median, a green arrowhead representing the mean, and whiskers extending from the box on either end representing the range of values observed. Statistic outliers are plotted individually using the ‘o’ symbol.

Comparing the different algorithms, we observe immediately from Supplementary Figure 4 that each of our greedy algorithms displays significantly lower gain than the random assignments on average, which demonstrates the usefulness of our anti-collusion schemes in reducing the collusion gains. Second, the low average collusion gain exhibited by the optimal solution computed by ILP on average validates our approach for minimizing the gain from collusion. Third, MMM-CGS and CGS approximated the minimum gain computed by ILP well, highlighting the effectiveness of the greedy algorithms in practice.

Comparing the optimized solutions computed under different settings, it is easy to observe that optimized average collusion gain (with the optimal ILP algorithm for example) does not necessarily correlate to the permutation space size; e.g., (5, 3, 2)[1] and (5, 3, 3) have the permutation space of the same size but different optimal gains; from (10, 3, 2) to (10, 5, 3), the size of the permutation space increased but the optimal gains decreased; and from (10, 5, 3) to (10,

[1]* $N = 5, M_2 = 3, M_1 = 2$ is noted as (5, 3, 2) for brevity.

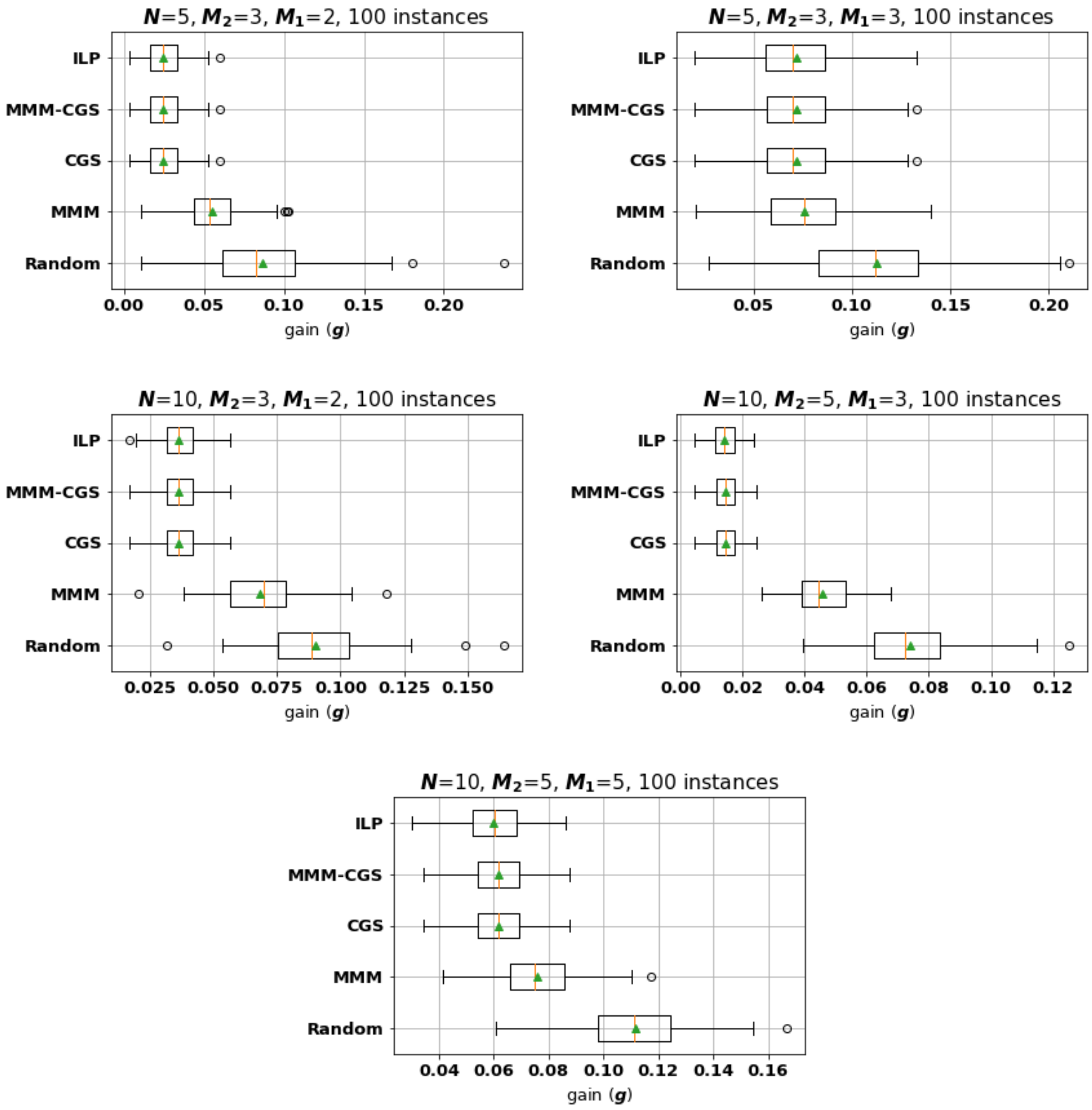

Supplementary Figure 4: Comparison of the greedy algorithms with the ILP method in terms of the average collusion gains.

5, 5), the size of the permutation space increased but the optimal gains increased. However, the optimized collusion gains do correlate to the difference between $M_2 - M_1$ (which has been implied by Theorem 1); i.e., an increased $M_2 - M_1$ value significantly reduces the collusion gain as demonstrated by (5, 3 ,3) versus (5, 3, 2), as well as (10, 5, 5) and (10, 3, 2) versus (10, 5, 3). In addition, increasing the number of students can also raise the collusion gain as shown in the case of (5, 3, 2) versus (10, 3, 2).

For each of the settings presented in Supplementary Figure 4, the ILP solution has very low collusion gain on average, validating our approach of suppressing collusion by question assignment without proctoring. Surprisingly, the CGS solution matches the minimum possible gain as obtained by ILP in over 95 out of a 100 instances in the settings of (5, 3, 2), (5, 3, 3) and (10, 3, 2), and matches the gain of ILP over 65 out of 100 instances in the settings of (10, 5, 3) and (10, 5, 5), validating the usefulness of our greedy heuristic algorithms. Additionally, the simple MMM-CGS algorithm dominates the other heuristic algorithms, but does not significantly outperform CGS in our experiments, which performs close to optimally, even thought its search space is restricted to the set of circular sequences. It appears that MMM which is initialized by a random assignment is sensitive to the initial assignment, and is prone to being stuck in locally optimal solutions, but still significantly outperforms random assignments.

Our experiments provide two key takeaways for educators: For a given number of students N: (1) The minimum collusion gain that can be obtained by our approach (eg. the ILP solution) is determined by the permutation space available to assign sufficiently different sets of questions and in orderings that minimize collusion, which can be controlled by the either increasing the size M_2 of the pool of questions, or decreasing the number of questions on each students' sequence M_1. (2) Greedy heuristic algorithms significantly outperform random assignments, and often approach the minimum possible average collusion gain in settings with a small permutation space for question assignment.

In Supplementary Figure 5, we compare our greedy algorithms on relatively large instances with $N = 100$ students, a pool of $M_2 = 30$ questions, and between $M_1 = 10$ and 20 questions per student. Our experiments demonstrate again that (1) our greedy algorithms significantly outperform random assignment, and (2) MMM-CGS and CGS significantly outperforms MMM initialized with random assignments.

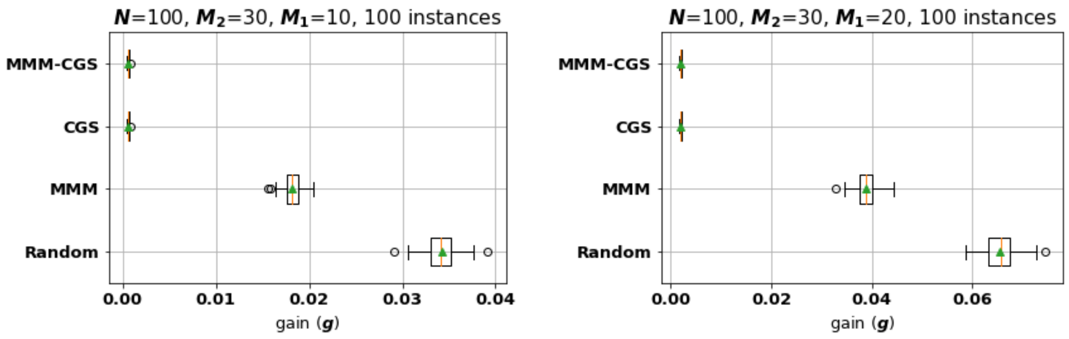

Supplementary Figure 5: Comparison of the average gain of greedy algorithms on large instances.

4 Final Exam Design

On April 28, 2020, 78 out of 85 undergraduate students in two classes separately taught by two instructors took the final exam of an undergraduate imaging course according to our optimized design [1]. The course is based on a standard textbook [2], with all video lectures available online [3] complemented with online lecture notes [4].

The class itself is divided in two main parts, fundamentals and imaging. Fundamentals cover measurements, linear systems, convolution, Fourier analyses, basic signal processing techniques such as filtering and sampling, basic imaging definitions, and measuring tests accuracy. Imaging covers medical imaging modalities; x-ray, computed tomography (CT), nuclear imaging such as PET (Positron Emission Tomography) and SPECT (Single Photon Emission Computed Tomography), Magnetic Resonance Imaging (MRI), ultrasound, and optical techniques such as microscopy and optical coherence tomography (OCT). The final exam covered signal processing and all imaging modalities. From this, a pool of multiple choice questions was created, each question with four options. The questions tested the main concepts of the class subjects and were similar in length since they all had equal grade points and a time period of two minutes each to answer. The number of questions was proportional to the materials taught in class, i.e., 20% of the questions covered x-ray and CT combined. Similarly, nuclear imaging, MRI, and ultrasound, each was the subject of 20% of the questions. 10% of the questions were about optical techniques and the remaining 10% covered basic imaging definitions, and measuring tests accuracy.

The questions included a mix of text, formulas, and figures, and were designed for open-book tests. To simplify the testing platform and add the difficulty in direct online searching of questions, all questions were included in the exam as images, and the students have four boxes labeled A to D to choose from by clicking on the desired option. The students could change their answer within the time period allocated for every question, but could not make changes afterwards. The length of the time window was empirically adjusted so that it is enough for high-competence students to finish the question comfortably but insufficient for unprepared students to search the answer without a good understanding of the content.

For the final exam, a pool of 80 questions was created, 60 of which were used, i.e., $M_2 = 60$. The remaining 20 were for students who requested a makeup exam. The exam consisted of 40 questions ($M_1 = 40$). Therefore, not all students were tested using the same 40 questions. Additionally, students were asked to join a WebEx video conference session with their respective instructor for questions or technical difficulties, which also served as a simple online proctoring. Students also need to log into our DOT platform with their RCS ID and RIN (unique IDs assigned to each student by our institute) to attend the exam. The identities of students were double-checked through the video by the instructor.

4.1 Sequence Assignment

Based on our anti-collusion scheme, an optimized assignment of the final exam $N = 85$, $M_2 = 60$, $M_1 = 40$, $Q = 4$ was first designed by GAS and then refined with our heuristic CGS algorithm.

4.1.1 Competence Estimation

The students' competences are estimated with their performance in the mid-term exam before the social distancing. The two classes were taught by different instructors, and have different mid-exams, but they will take the same final at the same time. Thus, their relative performances in the class were treated as their competence score rather than their real scores. The grades distribution of two classes were first normalized to the distribution with zero mean and unit standard deviation, and then combined together. It is worth mentioning that the students did not participate the mid-term exam were picked out before the normalization procedure, and then put back to the combined profile with 0 (using the averaged performance to estimate their performance). Finally, combined normalized grades were then linearly transformed to the range $[0.25, 1)$ to form the prior knowledge of the competence profile Y of the combined set of the students.

4.1.2 Colluding Matrix Construction

To perform the optimization, we heuristically construct a colluding matrix P depicting the probability of every student cheating from another student. Following the notations in main text, reasonable assumptions about colluding mechanisms are made as follows: (1) The probability of student i actively cheating is related to his/her competence y_i; Student 1 tends not to cheat since he/she could obtain no gain (risk greater than benefit), while student N will try all means to cheat since he/she will always gain (benefit greater than risk); (2) The probability of colluding happens between two students A and B is related to the difference of y_A and y_B. Student i will have the strongest willingness to cheat from student 1, but the least willingness to cheat from student j if $y_i = y_j$ since he/she cannot trust j more than himself/herself, and he/she will never cheat from j if $y_i > y_j$.

Based on the assumptions above, the colluding matrix P is heuristically constructed as follows:

$$p_{j,i} = \begin{cases} 0, & y_j \leq y_i \\ \frac{y_j - y_i}{\sum_{k=1}^{n_f(i)} (y_k - y_i)} (1 - p_{i,i}), & y_j > y_i \end{cases} \tag{S1}$$

$$p_{i,i} = \left[1 - \frac{\sum_{k=1}^{n_f(i)} (y_k - y_i)}{\sum_{k=1}^{N} (y_k - y_N)} \right]^{\eta} \tag{S2}$$

where $n_f(i)$ is defined as the number of elements in Y that are greater than y_i, and η is a non-negative constant which can be used to adjust students' willingness to cheat. Larger η will

increase the colluding probability, and students are supposed to always commit active cheating if $\eta = \infty$. Eqs. (S2) and (S1) define the probabilities of the cheating and non-cheating states of student i respectively, and in the cheating state, the possibility of student i will cheat from student j is proportional to their competence difference $y_j - y_i$ normalized by the sum of competence differences in all possible cases.

We further assume that students have different competences ($y_1 > y_2 > \ldots > y_N$), without losing generality (due to the fact that adding tiny differences to two equal y negligibly affects the result of g), we simplify the expression of $n_f(i)$ as

$$n_f(i) = i - 1 \tag{S3}$$

Hence, p_{ji} can be written more explicitly as follows:

$$p_{j,i} = \begin{cases} 0, & j < i \\ (1 - p_{i,i})(y_j - y_i)/(\sum_{k=1}^{i} y_k - i y_i), & j > i \\ \left[1 - \sum_{k=1}^{i} (y_k - y_i) / \sum_{k=1}^{N} (y_k - y_N)\right]^{\eta}, & j = i \end{cases} \tag{S4}$$

Note that this heuristic colluding matrix P may not exactly match real life but it is a reasonable start for optimization. In our construction, P puts a larger weight on the collusion between students with a larger competence difference than that with a small competence difference, which helps limit the collusion gain in the worst case. Since mismatches are very likely to exist between the model and the practice, the worst-case analysis needs to be performed on the optimized result. If the collusion gain calculated in the worst situation for the output assignment is not acceptable, the result should be used with caution or just use different initializations to generate diverse solutions and pick the best one.

4.1.3 Optimization Results

After optimization with CGS, the average collusion gain was reduced to 0.0073%, with the worst case collusion gain and the maximum individual collusion gain as 0.91% and 6.88% respectively, and the distribution of individual collusion gain in the worst case is shown in Supplementary Figure 6. From the figure, it can be seen that 90% students holds a maximum possible collusion gain below 2% while the others sparsely range from 3% to 7%, suggesting this is a practically good result.

Besides the anti-collusion feature, the nature of circular shifting sequences enabled us to ensure that every student can receive the same number of questions from the same lecture, i.e, every student shares 2 questions in Digital Signal Processing, 8 questions in CT, 7 questions in nuclear imaging, 5 questions in optics, 7 questions in MRI and 4 general questions. The ratio of questions in different topics can be arbitrarily adjusted. This feature could be easily achieved by arranging the positions of questions in the question bank with a proper pattern. With this feature, the exam could better measure the students' mastering of knowledge in different topics, and hence the instructors can obtain a well-rounded feedback with the exam.

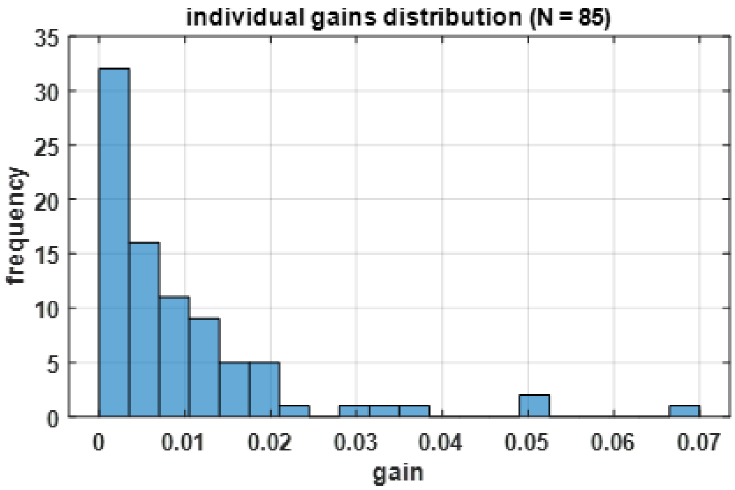

Supplementary Figure 6: The distribution of individual collusion gains in the worst case of the assignment after optimization ($N = 85, M_2 = 60, M_1 = 40, Q = 4$).

4.2 Robustness Relative to Noisy Y

The proposed general anti-collusion scheme works very well in terms of the maximum individual collusion gain in the simulation. To be mentioned, this does not rely on accurate student competence profiling. It can work well even with only the rank of the students' competences, and control the collusion gain to a desired level, as shown in the proof of Theorem 1 that only the ranking information has been used for implementing the scheme.

During the design of the exam, one would ask how robust of our method on the students' competences data with noises, since we need to infer Y from students previous performance, and randomness will inevitably make the Y noisy. In principle, it should be robust even if there are noises in the competence data. This can be readily understood that small noises will only make few students across the interval boundaries. The down-dropping student (DDS) could increase the maximum individual collusion gain g_{MI} since other students in the augmented group can cheat from the DDS, and the increment in g_{MI} will be no larger than the noise magnitude. Clearly, this will increase the worst case g_W due to the fact that all students in the same group will gain benefits. On the other hand, students in the upper group but with lower competences than the DDS can potentially cheat with the DDS which creates the inter-group collusion gains but this collusion gain is negligible in terms of g_W and g_{MI} since this inter-group gain should be much smaller than their maximum intra-group gains. As for the Up-floating case, only the up-floating student (UFS) will obtain an increased collusion gain through intra-group collusion but again the increment in g_{MI} should be smaller than the noise magnitude. The UFS will also benefit from inter-group collusion but the gain is much smaller than his/her intra-group collusion gain. The increment in g_{MI} will be smaller than the magnitude of noise. Thus, the grouping-based anti-collusion scheme should be robust against noise in students' competences.

5 Statistical Testing: Did Significant Collusion Occur

To assess whether the optimized DOT approach resulted in significant collusion, we formulated the hypotheses for aspects (i) and (ii). For testing the hypothesis for aspect (ii), there is no difference in the average number of correct answers for the first and the last 20 questions, we utilized the Wilcoxon signed-rank test, which is a standard non-parametric hypothesis test. To formulate a hypothesis for aspect (i), we considered that significant collusion did occur. This section provides details on how we tested this hypothesis, which is based on examining cases for which pairs of students gave the same answer to particular questions.

The format of the final exam stipulated that the 78 students were divided into 22 groups. As each group received a different set of problems, our focus here is on assessing the potential for intra-group collusion. More precisely, we can examine abnormal trends within the exam results *a posteriori*. With this in mind, we designed the following test procedure that focused on the 17 groups that had at least 3 students. We started by selecting a random integer number between 1 to 17, therefore identifying a group randomly. Next, from the selected group, we randomly selected two students which we considered to have engaged in collusion. Finally, we randomly selected $5 \leq n_q \leq 40$ questions for which we assumed that collusion had occurred.

If the pair of students gave the same answer to one of the n_q problems, irrespective of whether the answer given is correct or not, we assigned a logic 0 to this case. Conversely, if the two students gave different answers to a particular problem, we assigned the label 1 to describe this. To test for significant collusion, we repeated the procedure laid out in the preceding paragraph in a Monte Carlo fashion [5], *i.e.* randomly selecting groups, student pairs and problems.

The next step is combining $5 \leq m_p \leq 30$ randomly selected student pairs. To construct a random variable, we define the indicator function

$$\mathcal{I}_j(i) = \begin{cases} 0 & \text{We consider the students cheated} \\ 1 & \text{The students did not cheat} \end{cases}$$

where the index $1 \leq i \leq n_q$ refers to a randomly selected question. The index $j \geq 1$ labels the set of m_p randomly selected student pairs. The random variable $\mathcal{X} = x_j$ then describes the sample mean for the jth set of m_p randomly selected student pairs, *i.e.* $0 \leq x_j = \frac{1}{n_q} \sum_{i=1}^{n_q} \mathcal{I}_j(i) \leq 1$. Finally, by selecting M, *e.g.* $M = 30$, the number of Monte-Carlo runs is $M \times n_q$.

Based on the above procedure, we translate aspect (i) that there was significant collusion into the following null hypothesis:

$$\begin{aligned} H_0 &: \mu_{\mathcal{X}} = 0 \qquad \text{There is significant collusion} \\ H_1 &: \mu_{\mathcal{X}} > 0 \qquad \text{The students did not cheat} \end{aligned}$$

To test the above hypothesis, we can utilize the random variable $\mathcal{X}$. Following from the well-known central limit theorem, $\mathcal{X}$ has an asymptotic normal distribution. Using the sample containing the M observations of the random variable $\mathcal{X} = x_j$, *i.e.* x_1, ..., x_M, we can determine

the value of the test statistic $T = t$, $t = \overline{x}/s$, where s is the sample mean. The test statistic T has a t-distribution with $M - 1$ degrees of freedom [6]. This allows computing the p-value for rejecting the null hypothesis if $p < \alpha$ with α being the significance of the test, selected to be $\alpha = 0.05$.

The final step is to repeat the above procedure a total of K times, which yields p_1, p_2, $\ldots$, p_k, $\ldots$, p_K. As advocated in [7], to statistically evaluate these p-values, we computed the adjusted p-values to adjust for the false discovery rate. This, in turn, allows determining the false discovery rate (FDR) threshold. A FDR threshold below the significance α implies that each of the adjusted p-values are below α. For $K = 100$ repetitions of this Monte-Carlo method, $5 \leq n_q \leq 20$ randomly selected problems (# Problems), $5 \leq m_p \leq 30$ randomly selected student pairs (# Comparisons), Figure 3 in the main text shows that the FDR threshold is below $\alpha = 0.05$.

Even the extreme cases, describing a set of 100 times 5 randomly selected student pairs (two students within the same group) shows that there is no case where we fail to reject the null hypothesis. We therefore reject the hypothesis that the students engaged in significant collusion. In sharp contrast, we cannot make any statistically sound judgment as to whether individuals have assisted each other in answering the 40 questions. More precisely, there is no empirical evidence to accept that significant collusion occurred, for instance using cellphones for taking pictures and sending text messages or sending short emails of answers to particular problems.

It is interesting to observe that the number of questions has a negligible effect on the false detection rate threshold. This was not expected, as we considered the case of occasional collusion (a smaller value of n_q, say 5) to be more likely than systematic collusion (a larger value of n_q that is closer to 40). Conversely, the increase in the false discovery rate threshold when reducing the number of student pairs m_p is expected. By decreasing the sample size, or m_p, the size of the acceptance region increases accordingly, which reduces the probability of rejecting an incorrect null hypothesis (Type II error).

Reducing the lower boundary for m_p from 5 is not advisable, as m_p constitutes the sample size. More precisely, we observed a reduction in the FDR threshold for $m_p = 3, 4$ compared to the values obtained for $m_p = 5$, which we attributed to the lack of statistical information in the small sample. A point of contention is the assumption that the average over $10 \leq n_q \leq 40$ is drawn from a normal distribution. To verify that this is a valid assumption, we utilized the Anderson-Darling test [8] to test whether each sample of m_p observations was drawn from a normal distribution. By accepting 10% of violations, we observed that violations arose for around 18% of cases, $n_q < 25$. In practice, the use of the t-tests over alternative standard non-parametric hypothesis tests if the assumption of normality is violated often yields satisfactory results, *i.e.* [9]. Moreover, we repeated the same testing procedure using the standard sign test [6], which produced similar result to that depicted in Figure 3.

6 DOT Platform

To implement our DOT technology, we developed a software system using Flask [10], a web application written in Python [11]. This prototyping framework supports real-time communications between a secured database system and a frontend user-friendly interface. PostgreSQL [12] was used to record the information from users, and all data between PostgreSQL and Flask were transmitted with Psycogn2 [13], a PostgreSQL database adapter library because typically, the Psycogn database adapter can handle multiple database requests simultaneously. Furthermore, we used Jinja [14] embedded in Flask as the frontend interface, which is a designer-friendly HTML language for web development in Python. Through this mediator, and connected to PostgreSQL in the web framework, DOT is capable of handling a large number of requests at the same time.

Several interface screenshots of this DOT Platform are shown as Supplementary Figures 7 to 11. The aforementioned online exam as well as the data collection was conducted on this DOT platform.

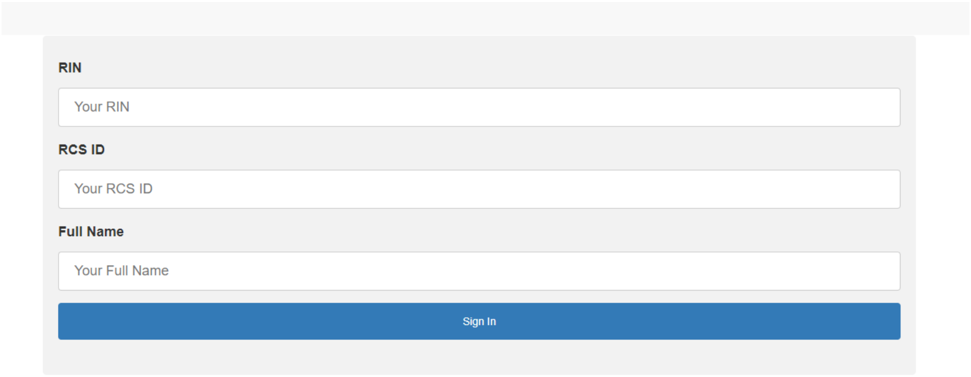

Supplementary Figure 7: The log in interface where students input their accounts and passwords to join the exam.

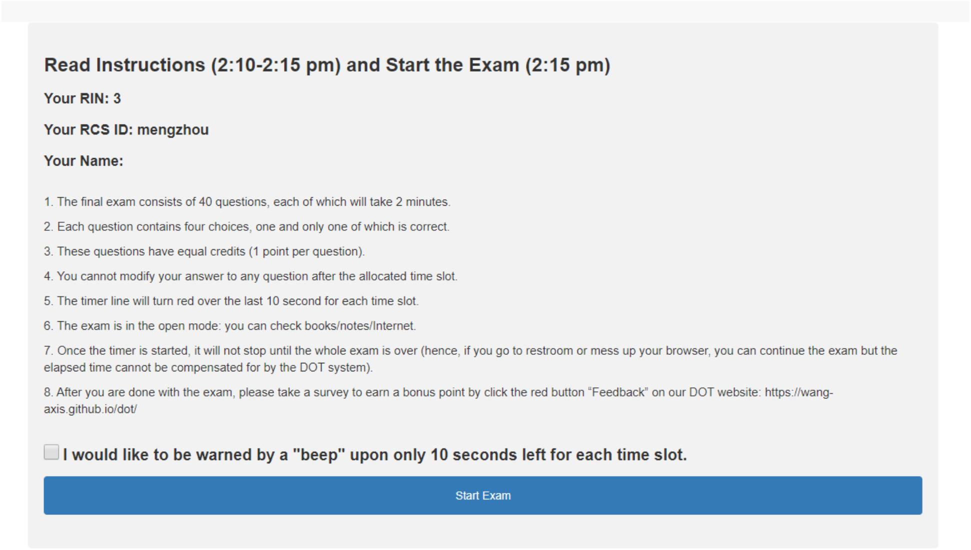

Supplementary Figure 8: The instruction interface displaying the general guidelines for the exam. This is right after students login, and students will read the guidelines and listen to the proctor' instructions waiting for the exam starts. Students can also choose whether to activate the beep function to remind them to put in their answers when there are only ten seconds left.

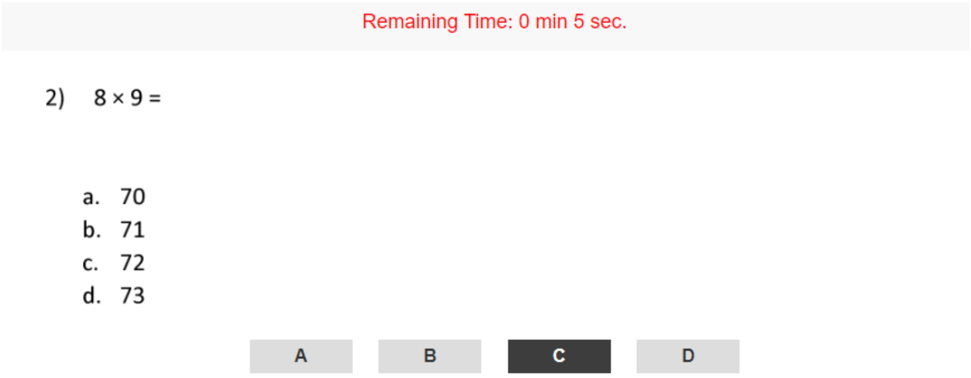

Supplementary Figure 9: The exam interface where questions are displayed. On the top of the questions, there is a timer counting down the left time allocated to this question. When the left time gets smaller than ten seconds, the timer will turn red to remind students to put in answers. If the student activates the beep function, he/she will also help a short beep when timer counts down to 10 seconds.

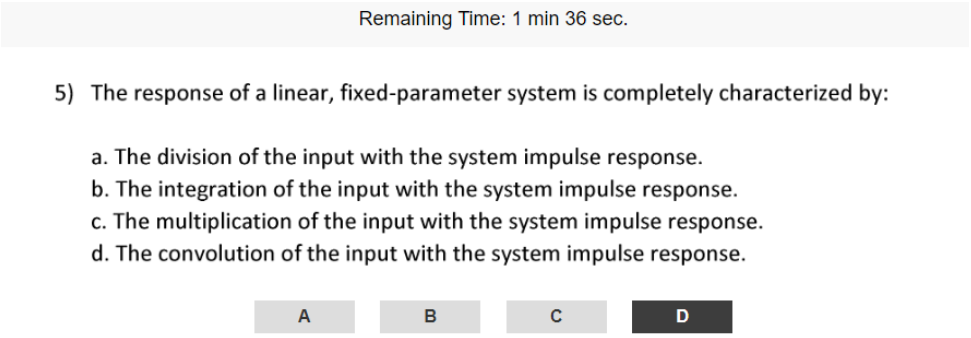

Supplementary Figure 10: The exam interface where questions are displayed. Students will have four boxes to click to indicate their choices of answers below each question.

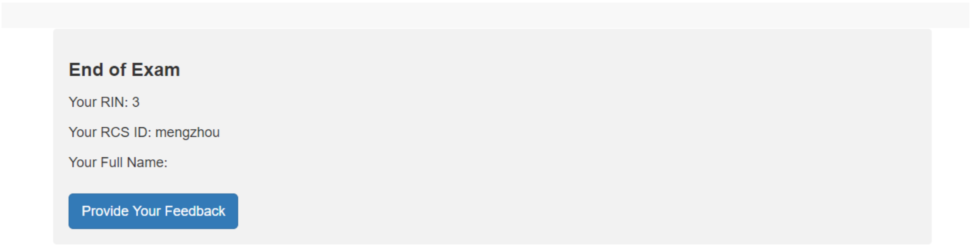

Supplementary Figure 11: The finish interface when a student finishes the exam (the exam period ends), he/she will be automatically directed to this interface to indicate the end of the exam. Students can also provide feedback by clicking the feedback bottom and answering a questionnaire

7 Random Sampling

As we mentioned in the Cyclic Greedy Searching Section, in the scenario without prior knowledge of students' competences, we prefer randomly assigning the students with random question sequences from the cyclic pool P_{CS} rather than from the permutation pool P_{SQ}. In this section, we calculate the expected collusion gain under randomly sampling from P_{CS} and random sampling from P_{SQ}, and prove that the former is more desirable (i.e. smaller expected collusion gain) than the latter.

Let us define an operation $EZ(\cdot)$ on a SQ pool S which calculates the expectation of $Fz(s_1, s_2)$ where s_1 and s_2 are two randomly selected elements from S. In other words, $EZ(S) = \text{mean}\{Z(S)\}$, where Z(S) is the positional matrix of S with diagonals set to the length of a sequence element from S, if replacement is allowed; otherwise, $EZ(S)$ equals to the mean of non-diagonal elements of $Z(S)$.

Theorem 2. *Suppose we randomly sample m different sequences from a SQ pool S (i.e. without replacement) and form $V = \{v_i \in S|, i = 1, 2, \ldots, m\}$ where $2 \leq m \leq |S|$. Then, $EZ(V) = EZ(S)$.*

Proof. If we first randomly select two sequences v_1 and v_2 from S, by definition, we have

$$E(Fz(s_1, s_2)) = E(Fz(s_2, s_1)) = EZ(S). \tag{S5}$$

Then we can continue to randomly select the rest sequences from the rest of S, but this does not affect the result we already have shown in Equation (S5). On the other hand, these two steps can be taken as one step that we randomly select m different sequences from S, hence, we cannot differentiate the m sequences from each other. In that sense, the expectation of Fz between any two of them should be the same. Combining with Equation (S5), we have

$$E(Fz(s_i, s_j)) = E(Fz(s_j, s_i)) = EZ(S), \tag{S6}$$

for $i, j = 1, 2, \ldots, m$, and $i \neq j$. Hence, the mean of the non-diagonal elements of the positional matrix of V equals to $EZ(S)$. This proves the theorem. ■

Theorem 3. *Suppose we randomly sample m sequences from a SQ pool S with replacement and form $V = \{v_i \in S|, i = 1, 2, \ldots, m\}$ where $2 \leq m \leq |S|$. Then, $EZ(V) = EZ(S)$.*

Proof. Follow the same idea of the proof of Theorem 2, but in one by one manner. ■

Theorem 4. *For any two M_1-length sequences s_1 and s_2 composed from the same M questions, $Fz(s_1, s_2) + Fz(s_2, s_1) = M_1 + n_{spos}$ where n_{spos} equals to the number of the questions appearing at the the same positions in s_1 and s_2.*

Proof. Let us denote the M questions as $[M_1] = \{1, 2, \ldots, M_1\}$. For the case that a question $i \in [M_1]$ appears at the same positions in s_1 and s_2, i contribute 1 to both $Fz(s_1, s_2)$ and $Fz(s_1, s_2)$. For the other case that a question $i \in [M_1]$ does not appears at the same positions in s_1 and s_2, i contribute 1 to either $Fz(s_1, s_2)$ or $Fz(s_1, s_2)$. There are M different questions in the sequences, hence, $Fz(s_1, s_2) + Fz(s_2, s_1) = M_1 + n_{spos}$. ■

Theorem 5. *For any two M_1-length sequences s_1 and s_2 that share n_{com} common questions, $Fz(s_1, s_2) + Fz(s_2, s_1) = n_{com} + n_{spos}$ where n_{spos} equals to the number of the questions appearing at the the same positions in s_1 and s_2.*

Proof. For unique questions only in s_1 or s_2, they do not contribute to neither $Fz(s_1, s_2)$ or $Fz(s_1, s_2)$. For the other two cases, we have the same conclusions as Theorem 4. There are only n_{com} questions contribute to $Fz(s_1, s_2) + Fz(s_2, s_1)$, hence, $Fz(s_1, s_2) + Fz(s_2, s_1) = n_{com} + n_{spos}$.

Now let us look at our problem, the notations remain the same as that in the Optimization Model Section. The expectation of collusion gain without prior knowledge of students' competences is the mean of the off-diagonal elements of the positional matrix of the assignment A, since all students are taken as identical and all collusion cases share the same weight. Theorems 2 and 3 tell us that the $EZ(\cdot)$ value of an assignments A generated by randomly sampling from a pool S should equal to $EZ(S)$, no matter of replacement is allowed or not. Hence, we have

$$E(g(A)) = EZ(S)N \tag{S7}$$

■

7.1 Circularly-shifting Pool

Now let us look at the cyclic sequence pool P_{CS} first, where no questions appear at the same position in any two sequences due to the nature of circular shifting.

7.1.1 $M_1 = M_2$

When replacement is allowed, we have

$$EZ(P_{CS}) = \frac{1}{M_2^2} \sum_{i,j=1}^{M_2} Z_{i,j}(P_{CS}) \tag{S8}$$

where $Z(P_{CS})$ is the positional matrix of P_{CS} and $Z_{i,i}(P_{CS}) = M_1$ for $i = 1, 2, \ldots, M_2$. Based on Theorem 4, it is easy to know that

$$Z_{i,j}(P_{CS}) + Z_{j,i}(P_{CS}) = M_1, \tag{S9}$$

holds for the off-diagonal elements. Hence, it is easy to obtain

$$EZ(P_{CS}) = \frac{1}{M_2^2}\left(\frac{M_2(M_2-1)}{2}M_1 + M_2M_1\right) = \frac{M_1+1}{2} \tag{S10}$$

Similarly, when replacement is not allowed, we have

$$EZ(P_{CS}) = \frac{1}{M_2(M_2-1)}\left[\sum_{i,j=1}^{M_2} Z_{i,j}(P_{CS}) - M_2M_1\right] = \frac{M_1}{2} \tag{S11}$$

7.1.2 $M_1 < M_2$

The definition of $EZ(\cdot)$ reminds us that the mean of the positional matrix of a sequence pool can be calculated by the expectation of Fz number between two randomly sampled sequences from the pool. Without the loss of generality, we can assume the first sequence is $s_{ref} = [1, 2, \ldots, M_1]$ for convenience, because for other cases, i.e., $[k_1, k_2, \ldots, k_{M_1}]$, we can relabel tag 1 to k_1, 2 to k_2, ..., M_1 to k_{M_1}. To be noted, by re-indexing, we have not changed the questions themselves.

For the case with replacement, s_{ref} can be combined with any sequence from P_{CS} with equal chance. If we list the sequences in P_{CS} in a right circular shifting manner started with s_{ref}, it is easy to find that for the second sequence s_i to be chosen from P_{CS}, where integer i indicates the position of the sequence in the sorted list of P_{CS},

$$Fz(s_{ref}, s_i) = \begin{cases} M_1 - i + 1, & i < M_1 + 1 \\ 0, & M_1 + 1 \leq M_2 \end{cases} \tag{S12}$$

To be mentioned, $s_i = s_{ref}$ for $i = 1$. Thus, we have the values of $Fz(s_{ref}, s_i)$ as $[M_1, M_1 - 1, \ldots, 1, 0, 0, \ldots, 0]$ for $i = 1, 2, \ldots, M_2$, with $M_2 - M_1$ zeros in total. Hence,

$$EZ(P_{CS}) = \frac{1}{M_2} \sum_{i=1}^{M_2} Fz(s_{ref}, s_i) = \frac{M_1(M_1 + 1)}{2M_2} \tag{S13}$$

Similarly for the case without replacement, the second choice can be only chosen from the rest of the pool, resulting $M_2 - 1$ choices (no s_{ref}). We have the values of $Fz(s_{ref}, s_i)$ as $[M_1 - 1, M_1 - 2, \ldots, 1, 0, 0, \ldots, 0]$ for $i = 2, 3, \ldots, M_2$, with $M_2 - M_1$ zeros in total. Hence,

$$EZ(P_{CS}) = \frac{1}{M_2 - 1} \sum_{i=2}^{M_2} Fz(s_{ref}, s_i) = \frac{M_1(M_1 - 1)}{2(M_2 - 1)} \tag{S14}$$

7.2 Complete Permutation Pool

Now let us look at the permutation pool P_{SQ}. Similar ideas can be followed to the calculation of $EZ(P_{CS})$ with the $M_1 < M_2$ case. $EZ(P_{SQ})$ can be calculated by the expectation of Fz number between two randomly sampled sequences from the pool. Similarly, we can assume the first sequence is $s_{ref} = [1, 2, \ldots, M_1]$ for convenience. Now we are going to calculate the mean Fz value of all the combinations of s_{ref} and any sequence in P_{SQ} for the case allowing replacement, and the mean Fz value of all the combinations of s_{ref} and the other sequences in P_{SQ} for the case without replacement.

Suppose the second sequence s_i has been chosen from P_{SQ}, and $s_i = [v_1, v_2, \ldots, v_{M_1}]$ where v_j for $j = 1, 2, \ldots, M_1$ are the question tags. Now that we have $s_{ref} = [1, 2, \ldots, M_1]$

and $s_i = [v_1, v_2, \ldots, v_{M_1}]$, it is easy to find that s_i can copy the problem v_j if $v_j \le j$, so an easy criterion can be formed to judge whether a question $v_j \in s_i$ contributes to $Fz(s_{ref}, s_i)$,

$$f(v_j) = \begin{cases} 1, & v_j \le j \\ 0, & v_j > j \end{cases} \tag{S15}$$

7.2.1 $M_1 = M_2$

Note that, by random permutation, we can generate much more question sequences than that obtained through circular shifting. P_{SQ} actually contains $M_2!$ elements. Suppose that s_i is a placeholder for a sequence, then the sum of all $Fz(s_{ref}, s_i)$ values can be calculated by question positions instead of by sequences, and for the case allowing replacement, it is

$$\text{sum}_{s_i \in P_{SQ}} Fz(s_{ref}, s_i) = \sum_{s_i \in P_{SQ}} \left[\sum_{j=1}^{M_1} f(v_j) \right] = \sum_{j=1}^{M_1} \left[\sum_{s_i \in P_{SQ}} f(v_j) \right]. \tag{S16}$$

and for the case not allowing replacement, it is

$$\text{sum}_{s_i \in P_{SQ}, i \ne 1} Fz(s_{ref}, s_i) = \sum_{s_i \in P_{SQ}, i \ne 1} \left[\sum_{j=1}^{M_1} f(v_j) \right] = \sum_{j=1}^{M_1} \left[\sum_{s_i \in P_{SQ}, i \ne 1} f(v_j) \right]. \tag{S17}$$

Each question from $\{1, 2, \ldots, M_2\}$ has an equal possibility to be v_j (the jth question in a sequence), and the frequency of each question to be the jth question all equals n/M_2 in P_{SQ}, where n is the number of sequences in P_{SQ} which is equal to $M_2!$. Thus, based on the criterion Equations (S15) and (S16) can be easily calculated with

$$\sum_{s_i \in P_{SQ}} f(v_j) = \frac{j}{M_2} n, \text{ for } j = 1, 2, \ldots, M_1 \tag{S18}$$

$$\text{sum}_{s_i \in P_{SQ}} Fz(s_{ref}, s_i) = n \sum_{j=1}^{M_1} \frac{j}{M_2} = \frac{n M_1 (M_1 + 1)}{2 M_2} = \frac{n(M_1 + 1)}{2} \tag{S19}$$

and Equation (S17) with

$$\text{sum}_{s_i \in P_{SQ}, i \ne 1} Fz(s_{ref}, s_i) = \text{sum}_{s_i \in P_{SQ}} Fz(s_{ref}, s_i) - Fz(s_{ref}, s_1) \tag{S20}$$

$$= \frac{n(M_1 + 1)}{2} - M_1 \tag{S21}$$

Hence, we can calculate the mean of all $Fz(s_{ref}, s_i)$ values by normalizing the results in Equations (S19) and (S20) with their corresponding number of cases, and obtain $EZ(P_{SQ})$ with and without replacement as follows:

$$EZ(P_{SQ}) = \frac{n(M_1 + 1)/2}{n} = \frac{M_1 + 1}{2} \tag{S22}$$

$$EZ(P_{SQ}) = \frac{n(M_1+1)/2 - M_1}{n-1} = \frac{M_1+1}{2} - \frac{M_1-1}{2(n-1)} \tag{S23}$$

7.2.2 $M_1 < M_2$

Similar calculations can be done with the case of $M_1 < M_2$, and the only changes are the size of P_{SQ} which $n = M_2!/(M_2 - M_1)!$ now and $M_2 \neq M_1$ now. Thus, we can easily obtain the results for the cases with and without replacement as

$$EZ(P_{SQ}) = \frac{nM_1(M_1+1)/2M_2}{n} = \frac{M_1(M_1+1)}{2M_2} \tag{S24}$$

$$EZ(P_{SQ}) = \frac{\frac{nM_1(M_1+1)}{2M_2} - M_1}{n-1} = \frac{n}{n-1}\frac{M_1(M_1+1)}{2M_2} - \frac{M_1}{n-1} \tag{S25}$$

7.3 Comparison between Pools

For the ease of comparison, the $EZ(\cdot)$ values of random assignments in those cases have been put in Supplementary Table 3. Based on Equation (S7), we know the expected average collusion gain of a random assignment actually equals to the $EZ(\cdot)$ value of the pool that the assignment has been sampled from, i.e., $E(g(A_{CS}))/N = EZ(P_{CS})$ and $E(g(A_{SQ}))/N = EZ(P_{SQ})$. Comparing the results in Supplementary Table 3, we can find for the random sampling with replacement, there is no difference between $EZ(P_{CS})$ and $EZ(P_{SQ})$ in both cases of $M_1 = M_2$ and $M_1 < M_2$. But for the random sampling without replacement, the results are little tricky. For the case $M_1 = M_2$ without replacement, it is easy to find that $EZ(P_{SQ})$ is always greater than $M_1/2$ except the equality at $M_1 = 2$. Since $n = M_2!$ increases rapidly, we have $EZ(P_{SQ}) \approx (M_1+1)/2$ and approximates to the value with replacement. Hence, in this case ($M_1 = M_2$ without replacement), random sampling from P_{CS} is better than from P_{SQ}. For the other case without replacement, we scale the $EZ(P_{SQ})$ a little bit for easier analysis,

$$\begin{aligned}
EZ(P_{SQ}) &= \frac{n}{n-1}\frac{M_1(M_1+1)}{2M_2} - \frac{M_1}{n-1} && \text{(S26)}\\
&= \frac{n}{n-1}\frac{M_1}{2}\left(\frac{M_1+1}{M_2} - \frac{2}{n}\right) && \text{(S27)}\\
&> \frac{M_1}{2}\left(\frac{M_1+1}{M_2} - \frac{2}{n}\right) && \text{(S28)}\\
&= \frac{M_1}{2}\left(\frac{M_1-1}{M_2-1} + \frac{M_1+1}{M_2} - \frac{2}{n} - \frac{M_1-1}{M_2-1}\right) && \text{(S29)}\\
&= EZ(P_{CS}) + \frac{M_1}{2}\left(\frac{M_1+1}{M_2} - \frac{2}{n} - \frac{M_1-1}{M_2-1}\right) && \text{(S30)}
\end{aligned}$$

Supplementary Table 3: Comparison between $EZ(P_{CS})$ and $EZ(P_{SQ})$.

Conditions		$M_1 = M_2$	$M_1 < M_2, n = \frac{M_2!}{(M_2-M_1)!}$
$EZ(P_{CS})$	w/ replacement	$\frac{M_1+1}{2}$	$\frac{M_1(M_1+1)}{2M_2}$
	w/o replacement	$\frac{M_1}{2}$	$\frac{M_1(M_1-1)}{2(M_2-1)}$
$EZ(P_{SQ})$	w/ replacement	$\frac{M_1+1}{2}$	$\frac{M_1(M_1+1)}{2M_2}$
	w/o replacement	$\frac{M_1+1}{2} - \frac{M_1-1}{2(n-1)}$	$\frac{n}{n-1}\frac{M_1(M_1+1)}{2M_2} - \frac{M_1}{n-1}$

The residual part in Equation (S30) can be proved to be non-negative when $M_2 \geq 3$ and $M_1 \geq 2$ as

$$\frac{M_1+1}{M_2} - \frac{2}{n} - \frac{M_1-1}{M_2-1} = \frac{2M_2-M_1-1}{M_2(M_2-1)} - \frac{2}{n} \tag{S31}$$

$$= \frac{2M_2-M_1-1}{M_2(M_2-1)} - \frac{2(M_2-M_1)!}{M_2!} \tag{S32}$$

$$\geq \frac{2M_2-M_1-1}{M_2(M_2-1)} - \frac{2(M_2-2)!}{M_2!} \tag{S33}$$

$$= \frac{1}{M_2(M_2-1)}[2M_2-M_1-1-2] \tag{S34}$$

$$= \frac{1}{M_2(M_2-1)}[M_2-M_1+M_2-3] \geq 0 \tag{S35}$$

Thus, we have $EZ(P_{SQ}) > EZ(P_{CS})$ when $M_2 \geq 3$ and $M_1 \geq 2$. It is easy to check that for the case $M_1 = 1$, $EZ(P_{SQ}) = EZ(P_{CS}) = 0$. Since $M_2 > M_1$, if $M_1 \geq 2$ then $M_2 \geq 3$. Therefore, we have $EZ(P_{SQ}) \geq EZ(P_{CS})$, and equality only holds on the situation $M_1 = 1$.

Overall, we always have $EZ(P_{SQ}) \geq EZ(P_{CS})$, and the equality only holds in the case with replacement or in the case with $M_2 = M_1 = 2$ or $M_2 > M_1 = 1$. Thus, sampling from the cyclic pool for random assignment tends to have a smaller expectation of the average collusion gain than sampling from the permutation pool, and should be a preferred choice if without sequence size issues.

References

[1] DOT, Distanced online testing, https://wang-axis.github.io/dot/ (2020).

[2] A. G. Webb, *Introduction to biomedical imaging* (John Wiley & Sons, 2017).

[3] G. Wang, Lecture, http://www.fully3d.org/rpi/ (2020).

[4] G. Wang, Lecture note, http://www.fully3d.org/rpi/assets/mile-v2-031818.pdf (2020).

[5] R. Y. Rubinstein, D. P. Kroese, *Simulation and the Monte Carlo Method* (John Wiley & Sons, 2016), third edn.

[6] D. C. Montgomery, G. C. Runger, *Applied Statistics and Probability for Engineers* (John Wiley & Sons, 2007), fourth edn.

[7] Y. Benjamini, Y. Hochberg, *Journal of the Royal Statistical Society: Series B (Methodological)* **57**, 289.

[8] T. W. Anderson, D. A. Darling, *Annals of Mathematical Statistics* **23**, 193.

[9] G. D. Ruxton, *Behavioral Ecology* **17**, 688–690.

[10] Flask, Flask, https://flask.palletsprojects.com/en/1.1.x/ (2020).

[11] Python, Python, https://www.python.org/about/ (2020).

[12] PostgreSQL, Postgresql, https://www.postgresql.org/ (2020).

[13] Psycogn2, Psycogn2, https://pypi.org/project/psycopg2/ (2020).

[14] Jinja, Jinja, https://jinja.palletsprojects.com/en/2.11.x/ (2020).
